# High‐Performance Ternary Organic Solar Cells via Isomeric Engineering of Nonfullerene Guest Acceptors

**DOI:** 10.1002/advs.202509661

**Published:** 2026-02-26

**Authors:** Zhenzhong Pan, Renjie Xu, Yuanyuan Jiang, Guangliu Ran, Kerui Liu, Wenkai Zhang, Meisu Zhou, Liheng Feng, Xiaozhang Zhu

**Affiliations:** ^1^ Institute of Applied Chemistry School of Chemistry and Chemical Engineering Shanxi University Taiyuan China; ^2^ Beijing National Laboratory for Molecular Sciences CAS Key Laboratory for Organic Solids Institute of Chemistry Chinese Academy of Sciences Beijing China; ^3^ School of Chemical Sciences University of Chinese Academy of Sciences Beijing China; ^4^ Department of Physics and Applied Optics Beijing Area Major Laboratory Center for Advanced Quantum Studies Beijing Normal University Beijing China; ^5^ Institute for Carbon‐Based Thin Film Electronics Peking University Taiyuan China

**Keywords:** isomeric effect, nanomorphology, nonfullerene electron acceptors, power conversion efficiency, ternary organic solar cells

## Abstract

Constructing ternary blends has been considered as a feasible approach to achieve highly efficient organic solar cells; however, the delicate choice of the guest components still needs labor‐intensive and time‐consuming work. Herein, the isomeric effects of guest nonfullerene acceptor on the physicochemical and photovoltaic properties of ternary OSCs are systematically investigated. Two isomeric NFAs, BTP‐γ‐Br and BTP‐δ‐Br, with different bromine‐substituted sites on IC terminal groups were designed and synthesized. BTP‐δ‐Br exhibits a larger dipole moment with red‐shifted absorption and tends to form overagglomerations with strong self‐aggregation behavior, while BTP‐γ‐Br shows weaker crystallinity with face‐on molecular orientation. The single crystal of BTP‐γ‐Br revealed that a special C≡N…H noncovalent interaction between the adjacent molecules can enhance intermolecular interactions. The D18:BTP‐γ‐Br‐based binary OSCs delivered a reduced nonradiative energy loss (0.18 eV) and a high *V*_OC_ of 0.931 V. Moreover, with the addition of isomeric NFAs into the D18:N3 host system, the D18:BTP‐γ‐Br:N3‐based ternary blends exhibited improved crystallinity with clear fibril networks, thus enhancing the efficient exciton dissociation and charge transport. Consequently, a high PCE of 19.2% (certified 18.9%) was obtained for D18:BTP‐γ‐Br:N3‐based devices. These results imply that rational design of guest NFAs is an efficient route to construct high‐performance OSCs.

## Graphical abstract

Two isomeric nonfullerene acceptors, BTP‐γ‐Br and BTP‐δ‐Br, were designed to investigate their effects on the optoelectronic properties and photovoltaic performance of ternary blends. Incorporating BTP‐γ‐Br into the D18:N3 binary system can enhance film crystallinity and suppress charge recombination, achieving a high PCE of 19.2% and a certified *J*_SC_ of 28.1 mA cm^−^^2^ in D18:BTP‐γ‐Br:N3‐based ternary solar cells.

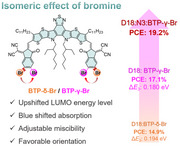

## 1 Introduction

Organic solar cells (OSCs) have made significant strides over the past decade, emerging as a promising alternative to conventional inorganic counterparts due to their lightweight, flexibility, and potential for cost‐effective, large‐scale production [1, 2, 3, 4, 5, 6, 7, 8]. OSCs generally adopted a bulk heterojunction structure to ensure efficient charge generation and transport, alleviating the bimolecular charge recombination due to the relatively low dielectric coefficient [9, 10, 11, 12, 13, 14]. To achieve an optimal polymer donor (D)‐nonfullerene acceptor (A) interpenetrated bicontinuous network, suitable crystalline phases with ordered molecular arrangement are required under the kinetic formation of thin film using spin‐coating technology [15, 16, 17, 18, 19]. In recent years, numerous approaches have been successfully devoted to fine‐tune nanomorphology, such as the design of novel materials [20, 21, 22, 23, 24, 25, 26, 27, 28], multicomponent strategies [29, 30, 31, 32, 33], solvent or additive engineering [34, 35, 36, 37], and the construction of pseudo‐planar heterojunction [38, 39, 40, 41, 42], and so on. Among them, ternary strategy via incorporating another functional third component into the binary host has been well‐demonstrated as a practical and potent avenue to construct highly‐efficient single junction solar cells, which not only fine‐tune the blend nanomorphology, but also delicately regulate the electronic properties of the materials, thus facilitating to deliver high short‐circuit density (*J*_SC_) and fill factor (FF) [29, 33, 43, 44, 45, 46, 47, 48, 49, 50]. Moreover, high open‐circuit voltage (*V*_OC_) with reduced nonradiative energy loss can be obtained by reducing electron‐vibration coupling with the dilution effect [46, 51, 52]. These merits of the ternary strategy have enabled single‐junction OSCs to realize PCEs of over 20% [14, 53, 54, 55]. However, despite these achievements, the construction of efficient ternary OSCs is labor‐intensive and time‐consuming with trial and error; therefore, deep insight into the intermolecular interactions between the guest component and the host binary system is urgently needed to establish the structure‐performance relationship, which will promote the further development of OSCs.

Fine‐tuning the intermolecular packing of non‐fullerene acceptors is a key approach to improve the nanomorphology of TOSCs [56, 57, 58]. The robust π‐π stacking interactions between the 2‐(3‐oxo‐2,3‐dihydro‐1H‐inden‐1‐ylidene)malononitrile (IC) terminal groups of A‐D‐A‐type NFAs resulted in the formation of well‐ordered, long‐range *J*‐aggregate structures, thereby establishing the primary electron transport pathways [59, 60]. The emergence of A‐DAD‐A‐type acceptors has facilitated the formation of distinct 3D molecular packing [24, 61, 62, 63]. This novel structural motif results in a substantial increase in intermolecular junctions, thereby enabling efficient electron hopping and isotropic charge transport, analogous to fullerene derivatives [64]. Isomerization refers to the introduction of structural variations in a molecule that result in different spatial arrangements of atoms, without altering the molecular formula. Minor structural perturbations, particularly within the central cores, alkyl/aryl side chains, and terminal groups of NFAs, can result in significant alterations to their photophysical properties [65, 66, 67, 68, 69, 70]. As a result, extensive research efforts have been dedicated to investigating these structural modifications. A series of isomeric NFAs, for example, ITIC and m‐ITIC [71], INIC1 and INIC2 [72], NBDT‐F_out_ and NBDT‐F_in_ [73], F‐2Cl, FEH2C8‐2Cl and F3EH‐2Cl [74], BTP‐ClBr, BTP‐ClBr1 and BTP‐ClBr2 [75], BTIC‐BO4Cl, BTIC‐BO4Cl‐βγ and BTIC‐BO4Cl‐βδ [76], S‐CSeF, A‐ISeF and A‐OSeF [77], were elaborately designed and synthesized. Therefore, employing an isomeric system offers a facile and pragmatic approach to investigating the intermolecular packing modes and aggregation behaviors of NFAs. Additionally, it also allows the stepwise modulation of NFAs’ frameworks, which will facilitate the systematic study of photovoltaic performance boosting.

Halogen atoms have been successfully demonstrated as a powerful tool to regulate the physicochemical and photovoltaic properties of NFAs [78, 79, 80]. In contrast to fluorine and chlorine atoms, bromine atoms, with a larger atomic radius of 0.114 nm and lower electronegativity (2.8), are more easily polarized due to their loosely held outermost electrons, facilitating the photoexciton separation [81]. Moreover, brominated compounds generally exhibited stronger crystallinity, which may help to induce more ordered molecular packing, thus improving charge transport [82]. To construct high‐performance single‐junction devices and systematically investigate the isomeric effects on ternary OSCs, herein, two nonfullerene acceptors, named as BTP‐γ‐Br and BTP‐δ‐Br, were designed and synthesized by introducing a monobromide atom at the γ‐ and δ‐position of the IC‐terminal unit, respectively. Compared to BTP‐δ‐Br, BTP‐γ‐Br exhibits slightly blue‐shifted light absorption, elevated LUMO energy level, and weaker crystallinity with face‐on molecular orientation. The molecular structure of BTP‐γ‐Br was well characterized by single crystal X‐ray diffraction analysis, in which a C≡N…H noncovalent interaction was observed between the adjacent molecules, which enhances the charge transport with electron mobility of (1.09 ± 0.09) × 10^−3^ cm^2^ V^−1^ s^−1^. By blending with polymer donor D18, BTP‐γ‐Br shows suitable surface and bulk morphology, while overagglomerates were distinctly observed in D18:BTP‐δ‐Br blends, deteriorating the charge dynamic properties. Consequently, D18:BTP‐γ‐Br binary device delivered a PCE of 17.1% with a high *V*_OC_ of 0.931 V and a low nonradiative energy loss of 0.18 eV, higher than that of D18:BTP‐δ‐Br (PCE = 14.9%, *V*_OC_ = 0.892 V, *E*_nr_ = 0.194 eV). In addition, BTP‐γ‐Br shows good miscibility with NFA N3, and the addition of BTP‐γ‐Br as a guest component into the D18:N3 host system can enhance the crystallinity of the blend film with clearly seen fibril nanonetworks, which facilitate the charge generation and dissociation. High power conversion efficiency of 19.2% (certified PCE of 18.9%) for D18:BTP‐γ‐Br:N3‐based ternary devices was achieved, which is much higher than that of D18:BTP‐δ‐Br:N3 blends with a PCE of 18.1%.

## 2 Results and Discussion

### 2.1 The Chemical and Optoelectronic Properties

**FIGURE 1:**
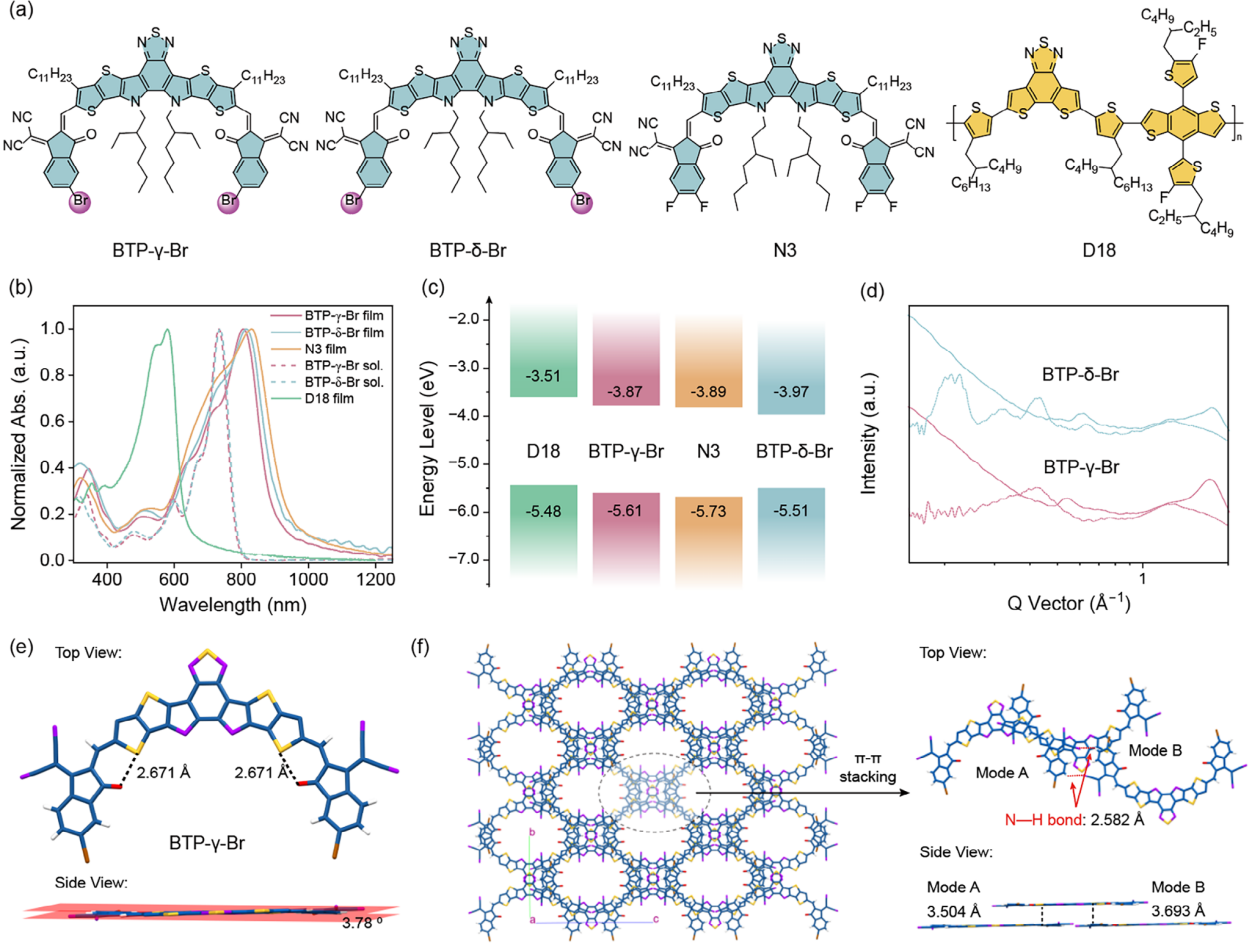
(a) The molecular structure of BTP‐γ‐Br, BTP‐δ‐Br, N3, and D18. (b) Normalized UV–vis–NIR absorption spectra of BTP‐γ‐Br, BTP‐δ‐Br, N3, and D18. (c) The energy level diagrams of BTP‐γ‐Br, BTP‐δ‐Br, N3, and D18 thin films. (d) The 1D GIWAXS diffraction profiles of BTP‐γ‐Br and BTP‐δ‐Br neat films in the in‐plane and out‐of‐plane directions. (e) The single crystal structure of BTP‐γ‐Br. (f) The intermolecular stacking diagram and the corresponding sets of dimers packing modes of BTP‐γ‐Br.

As shown in Figure 1a, the molecular structure of the two new isomeric nonfullerene acceptors BTP‐γ‐Br and BTP‐δ‐Br and N3, and the polymer donor D18 are depicted. BTP‐γ‐Br and BTP‐δ‐Br were synthesized by Knoevenagel condensation reaction between the dialdehyde compound **1** and 2‐(5‐bromo‐3‐oxo‐2,3‐dihydro‐1H‐inden‐1‐ylidene)malononitrile (IC‐γ‐Br) and 2‐(6‐bromo‐3‐oxo‐2,3‐dihydro‐1H‐inden‐1‐ylidene)malononitrile (IC‐δ‐Br), respectively. The two isomers were carefully characterized by ^1^H NMR, ^13^C NMR, and high‐resolution time‐of‐flight mass spectroscopy (HR‐TOFMS) as shown in the Supporting Information. All the compounds exhibited good thermal stability, and the decomposition temperature (5% weight loss) is 367 and 370°C for BTP‐γ‐Br and BTP‐δ‐Br, as revealed by thermogravimetric analysis (Figure S1).

Figure 1b shows the UV–vis–NIR absorption spectra of BTP‐γ‐Br, BTP‐δ‐Br, N3, and D18. The BTP‐γ‐Br and BTP‐δ‐Br solutions show almost the same absorption peaks at 735 nm, while the maximum absorption peak of the BTP‐γ‐Br thin film was located at 805 nm, which is blue‐shifted to 814 nm compared to the BTP‐δ‐Br thin film. However, BTP‐γ‐Br thin film exhibits a stronger light harvesting in the NIR region with a high absorption coefficient of 2.91 × 10^5^ cm^−1^ than that of BTP‐δ‐Br thin film (2.24 × 10^5^ cm^−1^) (Figure S2). The optical bandgap of BTP‐γ‐Br and BTP‐δ‐Br thin films is 1.36 eV and 1.33 eV, respectively, based on the onset absorption. The electrochemical properties of the two NFAs were checked by cyclic voltammetry (CV) measurements, and the CV curves and the corresponding energy level diagrams are shown in Figure S3 and Figure 1c. The highest occupied molecular orbital (HOMO) and the lowest unoccupied molecular orbital (LUMO) energy levels are −5.61/−3.87 eV for BTP‐γ‐Br film and −5.51/−3.97 eV for BTP‐δ‐Br film, respectively. The lower LUMO energy level of BTP‐δ‐Br is in agreement with the density functional theory (DFT) calculations, as shown in Figure S4. Contact angle measurements were conducted to investigate the miscibility between these components, and the corresponding results are presented in Figure S5 and Table S1. The surface free energy (γ) values are 35.70 mN m^−1^, 40.97 mN m^−1^, 42.06 mN m^−1^, and 39.46 mN m^−1^ for D18, BTP‐γ‐Br, BTP‐δ‐Br, and N3, respectively. According to the equation of:

$$\chi_{D-A}=K{\left(\sqrt{\gamma_{D}}-\sqrt{\gamma_{A}}\right)}^{2}$$

the Flory‐Huggins interaction parameters (χ) are 0.063*K* and 0.113*K* for the D18:BTP‐γ‐Br and D18:BTP‐δ‐Br systems, respectively. The lower χ value in the D18:BTP‐γ‐Br system indicates superior miscibility relative to the D18:BTP‐δ‐Br system. Furthermore, the wetting coefficients (ω) are calculated to analyse the distribution of the isomeric NFAs within the ternary blends [83]. As shown in Table S1, the ω_BTP‐γ‐Br_ and ω_BTP‐δ‐Br_ are ‐1.805 and ‐2.836, respectively, indicating that both isomeric NFAs tend to reside within the host acceptor phase when incorporated into the D18:N3 system.

In addition, the incorporation of a bromine atom at a different site shows a significant effect on NFA's polarizability. As shown in Figure S6, the dipole moments (µgs) of BTP‐γ‐Br and BTP‐δ‐Br are determined to be 2.39 and 3.26 Debye at ground state, respectively. Moreover, the quadrupole moments of BTP‐δ‐Br are larger than those of BTP‐γ‐Br in all directions, with the *Q*_ZZ_ component along the π–π stacking direction measuring 87.3 Debye and 74.2 Debye, respectively (Table S4). The larger dipole and quadrupole moments facilitate intermolecular interactions and enhance the crystallinity, while overagglomerates were formed in BTP‐δ‐Br films, leading to deteriorated photovoltaic performance as discussed below. Then, the excited state dipole moment (*µ*_e_) of NFAs was calculated using the time‐dependent DFT (TD‐DFT) method (Figure S6), and the difference dipole moment (Δµg_e_) between the excited and ground state was evaluated by the equation of

Δµg_e_ = $\sqrt{{\left(\mu_{gx}-\mu_{\mathrm{ex}}\right)}^{2}+{\left(\mu_{gy}-\mu_{\mathrm{ey}}\right)}^{2}+{\left(\mu_{gz}-\mu_{\mathrm{ez}}\right)}^{2}}$, in which µg*_x_*, µg_y_, µg_z,_ *and µ*_ex_, *µ*_ey_, *µ*_ez_ represent the dipole moment at ground and excited state in the x, y, and z axes, respectively. The calculated Δµg_e_s are 2.33 and 2.27 for BTP‐γ‐Br and BTP‐δ‐Br, respectively. The larger Δµg_e_ value implies more efficient intermolecular charge transfer with a reduced Coulombic binding energy of photoexcitons [84, 85, 86].

The thin film crystalline properties of the isomeric NFAs were further studied by the grazing‐incidence wide‐angle X‐ray scattering (GIWAXS). The 2D GIWAXS patterns and the corresponding line‐cuts of BTP‐γ‐Br and BTP‐δ‐Br are presented in Figures S7 and S8 and Figure 1d. The BTP‐γ‐Br thin film adopts obvious face‐on molecular orientation relative to the substrate, which can be demonstrated by the strong (010) diffraction peak in the out‐of‐plane (OOP) direction and (100) diffraction peak in the in‐plane (IP) direction. As summarized in Tables S2 and S3, the BTP‐γ‐Br neat film shows a (010) diffraction peak in the OOP direction at 1.679 Å^−1^, which corresponds to the π–π stacking distance of 3.74 Å and the crystal coherence length (CCL) of 27.7 Å. In the IP direction, the BTP‐γ‐Br neat film exhibits a (100) diffraction peak in the in‐plane (IP) direction at 0.422 Å^−1^, corresponding to the inter‐lamellar spacings of 14.9 Å and CCL of 75.4 Å. The BTP‐δ‐Br film shows a (010) diffraction peak in the OOP direction at 1.685 Å^−1^, corresponding to the π–π stacking distance of 3.73 Å and the CCL of 25.8 Å, and a (100) diffraction peak in the IP direction at 0.312 Å^−1^, corresponding to the π–π stacking distance of 20.1 Å and the CCL of 91.8 Å, respectively. The space charge limited current (SCLC) method was then used to evaluate the vertical charge transport properties of the NFAs with a device structure of ITO/ZnO/NFA/PDINN/Ag. As shown in Figures S9 and S10, the electron mobility was measured to be (1.09 ± 0.09) × 10^−3^ cm^2^ V^−1^ s^−1^ for BTP‐γ‐Br thin film, which is much higher than that of BTP‐δ‐Br film with electron mobility of (0.90 ± 0.02) × 10^−3^ cm^2^ V^−1^ s^−1^.

The single crystal of BTP‐γ‐Br (CCDC NO. 2450015) was further cultivated and analyzed to offer an insight into the molecular geometry and intermolecular packing modes. A BTP‐γ‐Br single crystal was obtained by using a solvent diffusion method in which methanol was slowly diffused into a BTP‐γ‐Br chloroform solution. The structure of BTP‐γ‐Br was measured by single‐crystal X‐ray diffraction (XRD) analysis, and the related crystallographic data were summarized in Table S5. The single crystal of BTP‐γ‐Br presents a monoclinic lattice with a I2/a space group. Figure 1e,f shows the molecular configuration and detailed interaction information. BTP‐γ‐Br shows a symmetric banana‐like molecular structure with an almost planar conjugated backbone, where the dihedral angle between the IC‐Br terminal group and the BTP core unit is 3.78°. The intramolecular noncovalent interaction between the oxygen atom at the IC‐Br group and the periphery sulfur atom of the BTP core was observed, and the distance of the S…O noncovalent bond is 2.671 Å, which is beneficial to maintain a planar backbone for BTP‐γ‐Br (Figure S10). The 3D network packing structure of BTP‐γ‐Br is formed by noncovalent interaction between those neighboring frameworks, wherein two categories of molecular packing modes exist. As shown in Figure 1f, mode A shows a large π–π stacking surface with dual intermolecular interaction between the IC‐Br terminal along the horizontal direction and the BTP core, while the IC‐Br terminal forms perpendicular intermolecular interaction with the BTP core in mode B. The corresponding π–π stacking distances are 3.504 Å and 3.693 Å, respectively, which are larger than those of other Y‐series derivatives because of the introduction of a larger bromine atom on the IC‐terminal. Interestingly, the intermolecular hydrogen bond was observed between the cyanogroup and the 6 position of the hydrogen atom between the adjacent IC‐Br terminal groups. The C≡N…H noncovalent interaction enables the adjacent molecule with a distance of 2.582 Å, which would facilitate the charge transport (Figure S11).

### 2.2 Photovoltaic Performance

**FIGURE 2:**
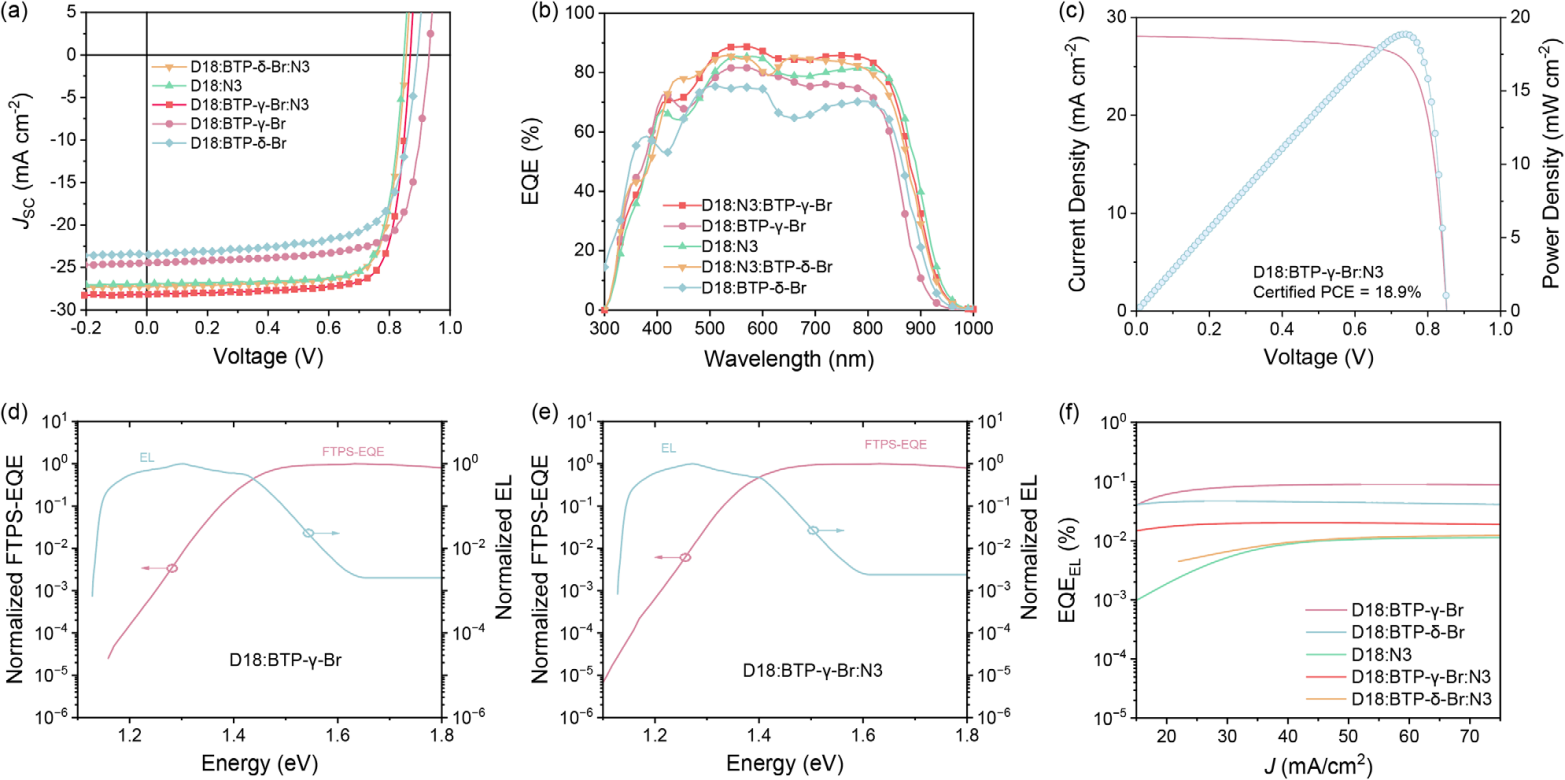
(a) *J*–*V* curves of the optimized binary and ternary photovoltaic devices under standard AM 1.5G irradiation (100 mW cm^−2^). (b) The corresponding EQE curves of the optimized OSCs. (c) Certified *J*–*V* curve and power density curve based on the optimized D18:N3:BTP‐γ‐Br device from the National Institute of Metrology (NIM), China. The semilogarithmic curves of normalized EL and FTPS‐EQE spectra of D18:BTP‐γ‐Br (d) and D18:N3:BTP‐γ‐Br (e)‐based OSCs. (f) EQE_EL_ curves of the optimal devices.

To investigate the isomeric effects on photovoltaic performance, both binary and ternary organic solar cells were fabricated with a normal device structure of ITO (indium‐tin oxide)/PEDOT:PSS (poly(3,4‐ethylenedioxythiophene):poly(styrene‐sulfonate))/D‐A blends/PDINN/Ag, in which PDINN was selected as the electron transporting layer to fine‐tune the work function of Ag cathode and reduce the interface contact resistance with photoactive layer. The corresponding current–voltage (*J‐*‐*V*) curves under standard AM 1.5G irradiation at 100 mW cm^−2^ are shown in Figure 2a, and the related device parameters are summarized in Table 1. The BTP‐γ‐Br‐based device delivers a higher *V*_OC_ of 0.931 V than that of the BTP‐δ‐Br‐based OSC (0.892 V) due to the slightly upshifted LUMO energy level and reduced energy loss as discussed below. Also, BTP‐γ‐Br‐based device presents a higher *J*_SC_ of 24.5 mA cm^−2^ and FF of 75%, suggesting a better blend nanomorphology with more favorable intermolecular packing and a suitable phase domain. Consequently, the isomer of BTP‐γ‐Br‐based OSCs offers a superior PCE of 17.1%, much higher than that of the D18:BTP‐δ‐Br‐based device (14.9%). The binary blends of D18:N3 were selected as the host system, and the corresponding devices delivered a decent PCE of 18.0% with a *V*_OC_ of 0.848 V, a *J*_SC_ of 26.9 mA/cm^2^, and a fill factor of 78.9%. The ternary OSCs of D18:N3:BTP‐γ‐Br were fabricated with an optimal weight ratio of 1:1.3:0.3 (w/w), and the devices gave the highest PCE of 19.2% with a *V*_OC_ of 0.869 V, a high *J*_SC_ of 28.2 mA/cm^2^, and a fill factor of 78.5%. With the addition of BTP‐δ‐Br as the third component into the D18:N3 host blend, the ternary OSCs of D18:N3:BTP‐δ‐Br exhibited a slightly improved *V*_OC_ and *J*_SC_, while the decreasing of FF in comparison with the host binary system. As a result, the optimal ternary devices of D18:N3:BTP‐δ‐Br exhibited a similar PCE of 18.1%, with a *V*_OC_ of 0.856 V, a high *J*_SC_ of 27.2 mA/cm^2^, and a fill factor of 77.7%. To further demonstrate the reliability of the excellent PCE of the devices, a certified efficiency of 18.9% was achieved for the optimal D18:N3:BTP‐γ‐Br‐based devices from the National Institute of Metrology, China (NIM), as shown in Figure 2c and Figure S12. Furthermore, Figure S13 and Table S6 demonstrate the versatility of the ternary strategy in PM6:Y6‐based system; the PM6:Y6:BTP‐γ‐Br‐based OSCs achieved a higher PCE of 18.8%.

**TABLE 1:** Summarized device parameters of OSCs based on D18:BTP‐δ‐Br, D18:BTP‐γ‐Br, D18:N3, D18:BTP‐δ‐Br:N3 and D18:BTP‐γ‐Br:N3 under AM 1.5G Illumination, 100 mW cm^−2^.

Blends	Ratio	*V*_OC_ (V)	*J*_SC_ (mA cm^−2^)	FF (%)	PCE (%)^a^	*E*_g_ (eV)	*E*_loss_ (eV)	Δ*E*_3_ ^c^ (eV)
D18:BTP‐γ‐Br	1:1.6	0.931	24.5	75.0	17.1 (17.0 ± 0.1)	1.430	0.499	0.180
D18:BTP‐δ‐Br	1:1.6	0.892	23.4	71.3	14.9 (14.7 ± 0.2)	1.406	0.514	0.194
D18:N3	1:1.6	0.848	26.9	78.9	18.0 (17.9 ± 0.2)	1.381	0.533	0.235
D18:N3:BTP‐γ‐Br	1:1.3:0.3	0.869	28.2	78.5	19.2 (19.0 ± 0.2)	1.396	0.527	0.215
D18:N3:BTP‐δ‐Br	1:1.3:0.3	0.856	27.2	77.7	18.1 (18.0 ± 0.1)	1.387	0.531	0.232
D18:N3:BTP‐γ‐Br^b^	1:1.3:0.3	0.855	28.1	78.8	18.9	—	—	—

^a^ Average PCEs in parentheses for 6 devices.

^b^ Certified PCE from the National Institute of Metrology, China (NIM).

^c^ The ΔE_3_ is calculated from the equation of ∆E_3_ = ‐*kT*ln(EQE_EL_).

To examine the properties of the photocurrent response of the devices, the corresponding external quantum efficiency (EQE) curves were recorded. As shown in Figure 2b, all the devices exhibited broad EQE response between 450 nm and 850 nm. For the BTP‐Br‐based binary OSCs, BTP‐δ‐Br‐based devices exhibit slightly red‐shifted absorption, which can be ascribed to the lower bandgap compared to the BTP‐γ‐Br‐based devices. Whereas, the BTP‐γ‐Br‐based devices show much higher EQE values in the range of 450–850 nm in comparison with the BTP‐δ‐Br‐based devices, contributing to achieving a superior *J*_SC_ value. The addition of BTP‐γ‐Br into the D18:N3 blend can further increase the EQE response in the range of 450–850 nm, and high EQE values of over 80% were obtained in the range of 490–830 nm. However, the EQE of ternary OSCs of D18:N3:BTP‐δ‐Br shows a negligible effect with the isomeric NFA BTP‐δ‐Br. By integrating the corresponding EQE curves, the calculated *J*_SC_ values are 23.6, 22.4, 26.0 mA/cm^2^ for the D18:BTP‐γ‐Br, D18:BTP‐δ‐Br, and D18:N3‐based binary OSCs, and 27.0, 26.2 mA/cm^2^ for the D18:N3:BTP‐γ‐Br and D18:N3:BTP‐δ‐Br‐based ternary OSCs, respectively. All the integrated *J*_SC_ values are consistent with the *J*_SC_s obtained from the corresponding J‐V curves, with a small deviation of less than 5%.

Different bromination sites of NFAs can significantly affect the open‐circuit voltage (*V*_OC_) in both binary and ternary blends, which prompts us to deeply understand the isomeric effects on the detailed energy loss (*E*_loss_). The detailed results of electroluminescence (EL) and Fourier‐transform photocurrent spectroscopy (FTPS) measurements were summarized in Table S6. The band gaps (*E*_g_s) of the blend films were determined from the EQE curves, and the calculated *E*_g_s are 1.430, 1.406, 1.381, 1.396, and 1.387 eV for D18:BTP‐γ‐Br, D18:BTP‐δ‐Br, and D18:N3, D18:N3:BTP‐γ‐Br, and D18:N3:BTP‐δ‐Br, respectively. The *E*_loss_s can be obtained with the equation of *E*_loss_ = *E*_g_ – *qV*_OC_, and the *E*_loss_ values are 0.499, 0.514, 0.533, 0.527, and 0.531 eV for D18:BTP‐γ‐Br, D18:BTP‐δ‐Br, D18:N3, D18:N3:BTP‐γ‐Br, and D18:N3:BTP‐δ‐Br, respectively. The detailed energy loss of solar cells can be divided into three components according to the Shockley‐Queisser (S‐Q) limit. The first component (Δ*E*_1_) is the energy loss caused by the radiative charge recombination above the bandgap, which is unavoidable, with values ranging from 0.25 V to 0.30 V. The Δ*E*_1_ values are calculated to be 0.268, 0.266, 0.264, 0.265, and 0.265 eV for the D18:BTP‐γ‐Br, D18:BTP‐δ‐Br, D18:N3, D18:N3:BTP‐γ‐Br, and D18:N3:BTP‐δ‐Br, respectively. The second component (Δ*E*_2_) is caused by the radiative energy loss below the bandgap, which is correlated with the band tail state. The calculated Δ*E*_2_ values are 0.051, 0.054, 0.034, 0.047, and 0.034 eV for D18:BTP‐γ‐Br, D18:BTP‐δ‐Br, and D18:N3, D18:N3:BTP‐γ‐Br, and D18:N3:BTP‐δ‐Br, respectively. The Urbach energy was also obtained by fitting the onset of the FTPS‐EQE spectra (Figure S14), and the calculated values are 27.8 ± 0.4, 26.1 ± 0.4, 23.9 ± 0.1, 25.0 ± 0.1, and 25.3 ± 0.1 meV for D18:BTP‐γ‐Br, D18:BTP‐δ‐Br, and D18:N3, D18:N3:BTP‐γ‐Br, and D18:N3:BTP‐δ‐Br, respectively. Last but not least, the third component (Δ*E*_3_) is derived from the non‐radiative charge recombination, which is one of the determining factors for OSCs to achieve highly efficient photovoltaic performance. According to the detailed balance theory, Δ*E*_3_ can be obtained by the equation of Δ*E*_3_ = ‐*k*Tln(EQE_EL_), in which the EQE_EL_ is the electroluminescence quantum efficiency at the injected current equal to the *J*_SC_ of the device. As shown in Figure 2f, the measured EQE_EL_ values are 8.20 × 10^−4^, 4.59 × 10^−4^, 0.91 × 10^−4^, 2.04 × 10^−4^, and 1.03 × 10^−4^ for D18:BTP‐γ‐Br, D18:BTP‐δ‐Br and D18:N3, D18:N3:BTP‐γ‐Br and D18:N3:BTP‐δ‐Br, respectively, and the corresponding Δ*E*_3_s were calculated to be 0.180 eV, 0.194 eV, 0.235 eV, 0.215 eV and 0.232 eV. The BTP‐γ‐Br‐based binary OSCs exhibit a lower Δ*E*_3_ of 0.180 eV than that of BTP‐δ‐Br‐based binary OSCs (0.194 eV). Besides, the incorporation of BTP‐γ‐Br into the D18:N3 system helps to reduce the Δ*E*_3_ value from 0.235 eV to 0.215 eV. These results indicate that the isomeric effect of brominated NFAs has a significant influence on the nonradiative recombination, and the introduction of BTP‐γ‐Br as the guest component can further suppress the host binary energy loss, thus improving the *V*_OC_s of the devices.

### 2.3 Charge Dynamics

**FIGURE 3:**
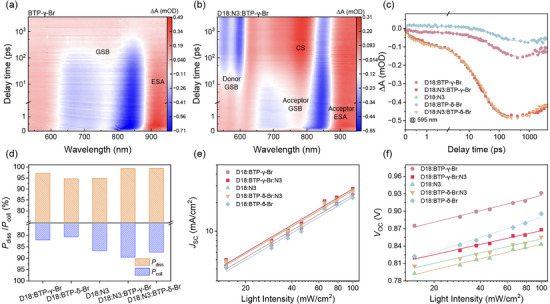
The femtosecond transient absorption spectra of BTP‐γ‐Br (a) and D18:N3:BTP‐δ‐Br (b) films. (c) The TA kinetics of the hole transfer process of the optimal blend films. (d) The *P_diss_* and *P*_coll_ of the optimal devices. (e) *J*_SC_ versus light intensity curves of the optimal devices. (f) *V*_OC_ versus light intensity curves of the optimal devices.

The carrier dynamics, including the charge generation (or exciton dissociation), transport, recombination, and extraction, have been investigated to get deep insight into the isomeric effect on the photovoltaic performance of the binary and ternary OSCs. The photoinduced hole transfer dynamics were first investigated by using the femtosecond transient absorption spectra (fs‐TA) technology. Figure 3a,b and Figures S15–S17 exhibit the color plots of fs‐TA spectra of the binary and ternary films, and the corresponding dynamic curves were plotted in Figure 3c. Considering the well‐separated absorption peaks of the donor and acceptor in the UV–vis spectroscopy, a low‐energy laser at 800 nm was selected as the pump beam to only excite the acceptor, which helps to investigate the hole transfer process from acceptor to donor. The negative‐going peaks at 700–850 nm appeared upon light illumination, and can be assigned as the ground state bleaching (GSB) signals of the acceptor. The hole transfer process from the NFA to the polymer donor D18 can be observed within ∼10 ps time scale with two new GSB peaks occurring at 540–610 nm, which is in line with the absorption feature of D18. We analyzed the hole transfer kinetics by using the GSB peak at 595 nm, and the corresponding curves were fitted by a double exponential function to quantitatively compare the hole transfer rate. The fitted values of τ_1_ and τ_2_ indicate the rate of photogenerated exciton separation at the D/A interfaces and the exciton diffusion process from the intradomain to the D/A interfaces, respectively. The binary blends of D18:BTP‐γ‐Br exhibit a τ_1_ value of 2.61 ± 0.61 ps, smaller than that of D18:BTP‐δ‐Br blends (8.18 ± 4.67 ps), implying more efficient charge separation in D18:BTP‐γ‐Br thin film. The τ_2_ values of D18:BTP‐γ‐Br and D18:BTP‐δ‐Br blends are 77.46 ± 3.23 ps and 95.22 ± 7.52 ps, respectively, suggesting the faster exciton diffusion process in D18:BTP‐γ‐Br thin film. The τ_1_ and τ_2_ values of D18:N3 host film are 1.78 ± 0.61 ps and 28.12 ± 0.53 ps, respectively. With addition of BTP‐γ‐Br into the host binary, D18:N3:BTP‐γ‐Br thin film exhibits smaller τ_1_ and τ_2_ values of 1.38 ± 0.10 ps and 26.60 ± 0.53 ps, while the corresponding values are 2.22 ± 0.17 ps and 33.98 ± 0.72 ps for D18:N3:BTP‐δ‐Br blend, indicating the introduction of BTP‐γ‐Br can improve the exciton diffusion and dissociation process, thus benefiting to suppress charge recombination and improving the *J*_SC_ and *V*_OC_.

We further studied the exciton generation, separation, and charge recombination properties of the corresponding devices to get insight into the variation of the photovoltaic performances. The relationship of the photocurrent density (*J*_Ph_) and the effective voltage (*V*_eff_) was investigated as shown in Figure 3d and Figure S18. For the D18:BTP‐γ‐Br binary device, the *J*_Ph_ increases with *V*_eff_ in the low effective voltage region, and *J*_Ph_ reaches a saturation state (*J*_sat_) with *V*_eff_ over 2.0 V, which implies the photoexcited excitons can be efficiently divided into free charges and collected by the electrodes. However, the D18:BTP‐δ‐Br binary device cannot reach the saturation current with *V*_eff_ over 3.0 V, which is consistent with the low FF. The exciton dissociation probability (*P*_diss_) and the charge collection probability (*P*_coll_) can be calculated by the equation of *P* = *J*/*J*_Sat_, in which the *J* represents the photocurrent density at short circuit and the maximum output power conditions, respectively. The *P*_diss_ values are estimated to be 97.1%, 94.7%, 94.8%, 99.3%, and 99.4% for D18:BTP‐γ‐Br, D18:BTP‐δ‐Br, D18:N3, D18:N3:BTP‐γ‐Br, and D18:N3:BTP‐δ‐Br, respectively, suggesting the more efficient exciton dissociation presented in BTP‐γ‐Br‐based binary and ternary devices. Moreover, the calculated *P*_coll_ values are 82.0% for D18:BTP‐γ‐Br, 80.6% for D18:BTP‐δ‐Br, 86.7% for D18:N3, 89.6% for D18:N3:BTP‐γ‐Br, and 87.4% for D18:N3:BTP‐δ‐Br, respectively. The higher *P*_diss_ and *P*_coll_ values for D18:N3:BTP‐γ‐Br‐based ternary OSCs suggest more efficient exciton dissociation and free charge extraction, thus facilitating the higher *J*_SC_ and FF.

The charge recombination process of the devices was then investigated by uncovering the variation of *J*–*V* curves upon different light intensities (*P*_light_) illumination. The linear relationship between *V*_OC_ and ln(*P*_light_) follows the equation of *V*_OC_ ∝ (*nkT*/*q*)ln(*P*_light_), where *k*, *T*, *q* are the Boltzmann constant, absolute temperature, and elementary charge, respectively, and the slope accounts for the dominant recombination mechanism of the devices (trap‐assisted Shockley‐Read‐Hall (SRH) monomolecular recombination or bimolecular recombination). As shown in Figure 3f, the *n* values are determined to be 1.16 for D18:BTP‐γ‐Br, 1.12 for D18:N3, 1.05 for D18:N3:BTP‐γ‐Br, and 1.07 for D18:N3:BTP‐δ‐Br, respectively, indicating that the trap‐assisted SRH recombination mechanism is well‐suppressed in those devices. However, the D18:BTP‐δ‐Br device exhibits a larger *n* value of 1.54, which can be attributed to its unfavorable nanomorphology and the associated more severe trap‐assisted recombination. The dependence of *J*_SC_ and *P*_light_ can be expressed as the power law *J*_SC_ ∝ *P*_light_ *^α^*, where *α* is the exponential factor. If the *α* value equals 1, the corresponding device exhibits negligible bimolecular recombination. As shown in Figure 3e, the calculated *α* values are 0.945, 0.945%, and 0.947, for D18:BTP‐γ‐Br, D18:BTP‐δ‐Br, and D18:N3, respectively. With the addition of BTP‐Br isomers into D18:N3 host films, the *α* value is increased to 0.954 for D18:N3:BTP‐γ‐Br, while D18:N3:BTP‐δ‐Br kept almost the same value (0.947) with D18:N3, which implies that bimolecular charge recombination can be efficiently suppressed in BTP‐γ‐Br‐based binary and ternary OSCs.

The charge transport properties of the binary and ternary devices were checked by using the space‐charge limited current (SCLC) method, as displayed in Figures S19–S21, and the corresponding data were summarized in Table S8. The carrier mobilities of the blends were measured with a hole‐only device structure of ITO/PEDOT:PSS/photoactive layer/Au for hole mobility and an electron‐only device structure of ITO/ZnO/photoactive layer/PDINN/Ag for electron mobility. The hole and electron mobilities were calculated to be (1.78 ± 0.13) × 10^−4^, (0.83 ± 0.02) × 10^−4^, (4.29 ± 0.45) × 10^−4^ and (6.53 ± 0.14) × 10^−4^, (6.25 ± 0.34) × 10^−4^, (9.37 ± 0.18) × 10^−4^ cm^2^ V^−1^ s^−1^ for D18:BTP‐γ‐Br, D18:BTP‐δ‐Br and D18:N3, with *µ*_e_/*µ*_h_ ratios of 0.27, 0.13, and 0.46, respectively. For the ternary blends, D18:N3:BTP‐γ‐Br exhibited higher hole and electron mobilities of (7.33 ± 0.32) × 10^−4^ and (8.43 ± 0.24) × 10^−4^ cm^2^ V^−1^ s^−1^, respectively, than that of D18:N3:BTP‐δ‐Br, (6.34 ± 0.54) × 10^−4^ and (8.07 ± 0.08) × 10^−4^ cm^2^ V^−1^ s^−1^. Over, BTP‐γ‐Br‐based binary and ternary devices exhibit more efficient hole transfer, charge separation, and transportation properties than those of BTP‐δ‐Br, which is consistent with the higher *J*_SC_s and FFs.

### 2.4 Morphology Analysis

**FIGURE 4:**
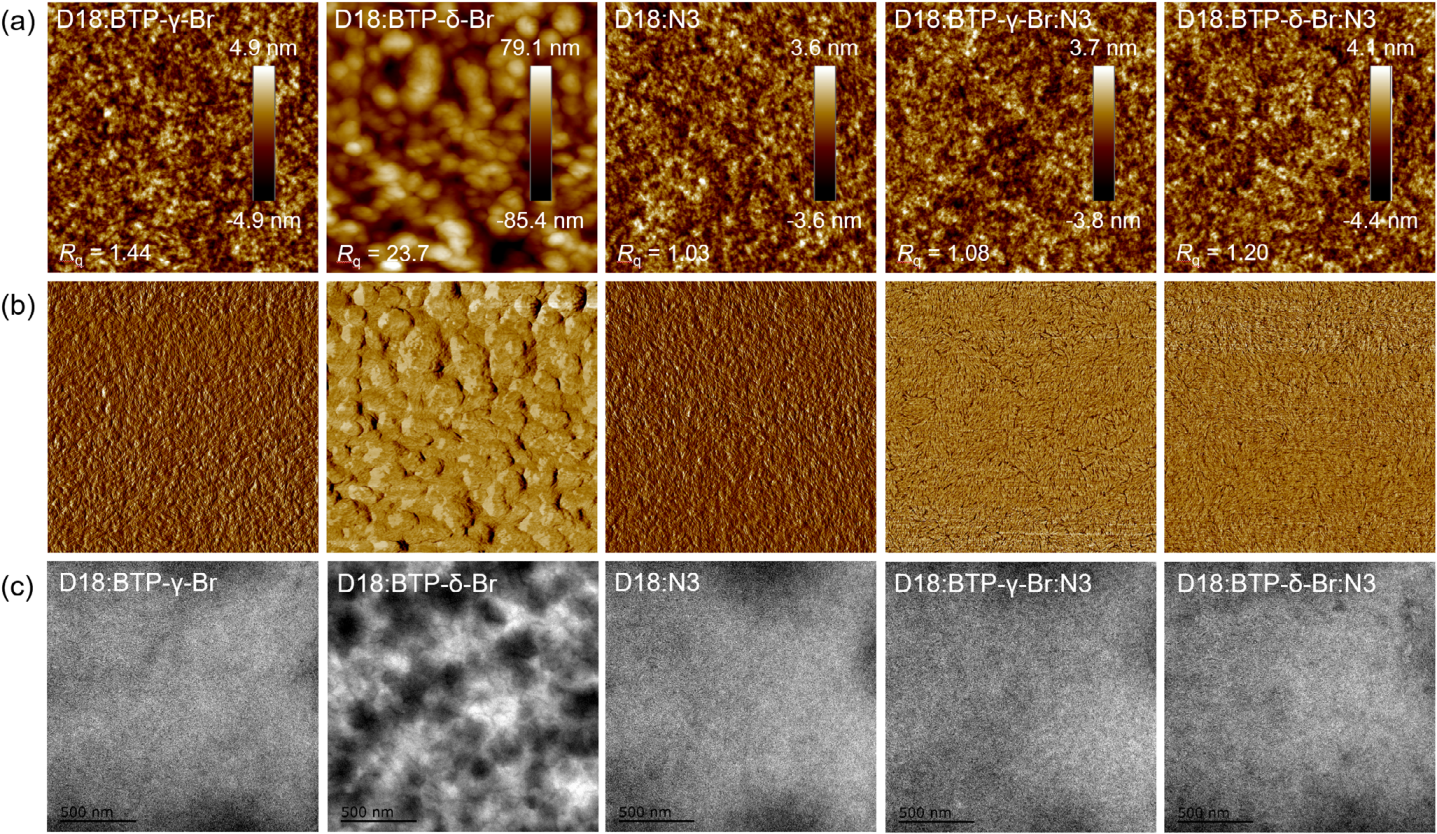
The AFM (2 × 2 um) height (a) and phase (b) images and the corresponding TEM (c) images for D18:BTP‐γ‐Br, D18:BTP‐δ‐Br, and D18:N3, D18:N3:BTP‐γ‐Br, and D18:N3:BTP‐δ‐Br.

To further illustrate the isomeric effects on photovoltaic performances of the binary and ternary devices, the atomic force microscopy (AFM) and transmission electron microscopy (TEM) were applied to analyse the top‐surface textures and bulk nanomorphology of the blend thin films. Figure 4a,b shows both the height and phase images of the blends. The D18:BTP‐γ‐Br binary blend exhibits a relatively smoother surface morphology with the root‐mean‐square roughness (*R*_q_) of 1.44 nm. However, the D18:BTP‐δ‐Br exhibits a much rougher surface with a *R*_q_ value of 23.7 nm, in which large overagglomerations were clearly seen in both height and phase images. The distinct surface morphology of the isomeric NFAs‐based blends may contribute to the discrepancy in photovoltaic performance. Comparatively, the addition of BTP‐γ‐Br and BTP‐δ‐Br into the D18:N3 host system results in slight variations in the nanomorphology of the ternary blends shows slight variations. As shown in Figure 4c, the TEM measurements further demonstrated the variation trend observed in AFM. All D18:BTP‐γ‐Br, D18:N3, and D18:N3:BTP‐γ‐Br blends exhibit homogeneous blend nanomorphology, while the D18:BTP‐δ‐Br blends exhibit large NFAs aggregations. The overagglomerated NFA domains may cause unfavorable charge recombination, leading to undesired *J*_SC_s and FFs.

**FIGURE 5:**
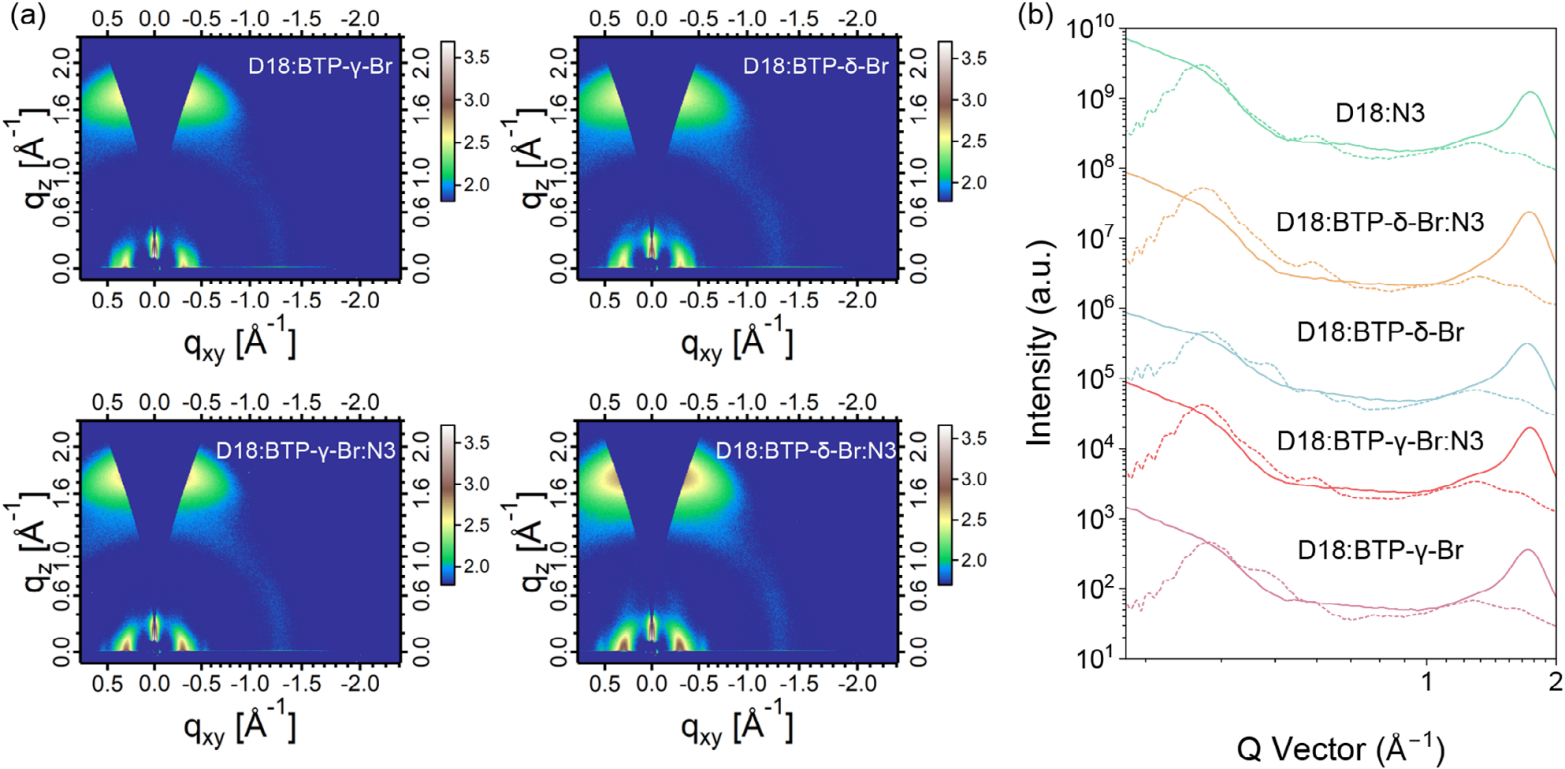
(a) 2D GIWAXS patterns of D18:BTP‐γ‐Br, D18:BTP‐δ‐Br, D18:N3:BTP‐γ‐Br, and D18:N3:BTP‐δ‐Br blend films. (b) The corresponding 1D line‐cuts in the in‐plane and out‐of‐plane directions.

2D GIWAXS measurements were conducted to analyze the isomeric effects of NFAs on molecular crystallization behavior and intermolecular packing features in blend films. Figure 5 exhibits the 2D GIWAXS patterns and the corresponding 1D line scattering curves, and the related data are listed in Tables S9 and S10. All the blends present distinct (010) diffraction peaks in the out‐of‐plane (OOP) direction, indicating the face‐on dominated molecular orientation. The locations of (010) peaks are 1.715 Å^−1^, 1.708 Å^−1^, 1.738 Å^−1^, 1.733 Å^−1^, and 1.729 Å^−1^ for D18:BTP‐γ‐Br, D18:BTP‐δ‐Br, and D18:N3, D18:N3:BTP‐γ‐Br, and D18:N3:BTP‐δ‐Br, respectively. The calculated π‐π stacking distances are 3.66 Å, 3.68 Å, 3.61 Å, 3.62 Å, and 3.63 Å for D18:BTP‐γ‐Br, D18:BTP‐δ‐Br, and D18:N3, D18:N3:BTP‐γ‐Br, and D18:N3:BTP‐δ‐Br, respectively, and the CCLs are 20.3 Å, 18.5 Å, Å 23.5 Å, 22.6 Å, and 22.2 Å. The addition of BTP‐γ‐Br can facilitate the crystallization of the host blend, thus improving intermolecular interaction and carrier mobilities, which is consistent with the SCLC measurements.

## 3 Conclusion

In summary, we have systematically investigated the isomeric effect of brominated nonfullerene acceptors on ternary organic solar cells, demonstrating that precise halogen positioning critically governs photovoltaic performance. Two isomeric NFAs, BTP‐γ‐Br and BTP‐δ‐Br with different bromine substitution sites at the IC‐terminal groups exhibited distinct properties: BTP‐γ‐Br displays blue‐shifted absorption, higher LUMO energy (−3.87 eV), and face‐on molecular packing due to a unique C≡N⋯H noncovalent interaction revealed by single‐crystal analysis, enhancing charge transport (*µ*_e_ = 1.09 × 10^−^^3^ cm^2^ V^−^^1^ s^−^^1^). In contrast, BTP‐δ‐Br forms overaggregates with red‐shifted absorption, leading to poor morphology and lower performance. Binary OSCs with D18:BTP‐γ‐Br achieve a PCE of 17.1% (vs. 14.9% for BTP‐δ‐Br), attributed to reduced nonradiative loss (0.18 eV) and higher *V*_OC_ (0.931 V). By incorporating BTP‐γ‐Br into D18:N3 further optimize the nanomorphology that enhances exciton dissociation and charge transport and suppresses nonradiative recombination, that improve photovoltage, yielding a record PCE of 19.2% (certified PCE of 18.9% from NIM, China). Our work underscores the pivotal role of halogen substitution geometry in tuning optoelectronic properties and aggregation behaviors, providing a design strategy for high‐efficiency OSCs via rational isomeric engineering. The findings highlight ternary systems with optimized guest NFAs as a viable route to minimize trial‐and‐error in the development of highly efficient OSCs.

## Conflicts of Interest

The authors declare no conflict of interest.

## Supporting information




**Supporting File**: advs74544‐sup‐0001‐SuppMat.docx.

## Data Availability

The data that support the findings of this study are available in the supplementary material of this article.
