# Supplementary material for: High‐Performance Ternary Organic Solar Cells via Isomeric Engineering of Nonfullerene Guest Acceptors
Source: Adv Sci (Weinh). 2026 Feb 26;13(25):e09661. doi: 10.1002/advs.202509661 (PMC13137825; doi:10.1002/advs.202509661)
Supplement: Supplementary file 1 — Supporting File: advs74544‐sup‐0001‐SuppMat.docx. [file ADVS-13-e09661-s001.docx]

Supporting Information

High-Performance Ternary Organic Solar Cells via Isomeric Engineering of Nonfullerene Guest Acceptors

Zhenzhong Pan, Renjie Xu, Yuanyuan Jiang, Guangliu Ran, Kerui Liu, Wenkai Zhang, Meisu Zhou,* Liheng Feng,* and Xiaozhang Zhu*

**Materials**

All the reactions dealing with air- or moisture-sensitive compounds were carried out in a dry reaction vessel under a positive pressure of nitrogen. Unless otherwise stated, starting materials were obtained from Adamas, Aldrich, and J&K and were used without any further purification. D18 was purchased from Hyper Inc. in Jiaxing, Zhejiang.

**Measurements**

Hydrogen nuclear magnetic resonance (^1^H NMR) and carbon nuclear magnetic resonance (^13^C NMR) spectra were measured on BRUKER DMX 400 spectrometers. Chemical shifts for hydrogens are reported in parts per million (ppm, δ scale) downfield from tetramethylsilane and are referenced to the residual protons in the NMR solvent (CDCl_3_:δ 7.26). ^13^C NMR spectra were recorded at 100 MHz. Chemical shifts for carbons are reported in parts per million (ppm, δ scale) downfield from tetramethylsilane and are referenced to the carbon resonance of the solvent (CDCl_3_:δ 77.0). The data are presented as follows:chemical shift, multiplicity (s = singlet, d = doublet, t = triplet, m = multiplet and/or multiple resonances, br = broad), coupling constant in Hertz (Hz), and integration. MALDI-TOF measurements were performed on an Applied Biosystems 4700 Proteomics Analyzer. Elemental analyses were measured on a Carlo Erba 1106 elemental analyzer. UV-vis spectra were recorded on a JASCO V-570 spectrometer. Cyclic voltammetry (CV) measurements were carried out on a CHI640C analyzer in a conventional three-electrode cell setup with glassy-carbon electrode as the working electrode, a platinum wire as the counter electrode, Ag/Ag^+^ as the reference electrode and calibrated with ferrocene/ferrocenium (Fc/Fc^+^) as an external potential marker in anhydrous CH_2_Cl_2_ solution containing 0.1 M tetrabutylammonium perchlorate (TBAP) as a supporting electrolyte under a nitrogen atmosphere at room temperature. All potentials were corrected against Fc/Fc^+^. CV was measured with a scan rate of 100 mV/s. Thermogravimetric analysis (TGA) was performed on a Shimadzu DTG 60 instrument at a heating rate of 10 °C min^−1^ under a N_2_ atmosphere with runs recorded from room temperature to 550 °C. EQE_EL_ values were obtained from an in-house-built system comprising a Hamamatsu silicon photodiode 1010B, a Keithley 2400 Source Meter for supplying voltages and recording injected current, and a Keithley 485 picoammeter for measuring the emitted light intensity. FTPS-EQE was measured using a Vertex 70 fro Bruker Optics, equipped with a quartz tungsten halogen lamp, quartz beam splitter and external detector option. A low-noise current amplifier (SR570) was used to amplify the photocurrent produced on illumination of the photovoltaic devices with light modulated by the Fourier transform infrared spectroscope (FTIR). The output voltage of the current amplifier was fed back into the external detector port of the FTIR, to be able to use the FTIR's software to collect the photocurrent spectrum.

**Fabrication of organic solar cells**

The devices were fabricated with a conventional structure of ITO/PEDOT:PSS/Active layer/PDINN/Ag. The ITO-coated glass substrates were cleaned with de-ionized water, acetone, and isopropyl alcohol in successive 20 min sonication steps, then applying a final 20 min oxygen plasma treatment to eliminate any remaining organic component before using. A thin layer (ca. 20 nm) of PEDOT:PSS was firstly spin-coated on the pre-cleaned ITO-coated glass substrates at 4000 rpm for 30 s and then baked at 150°C for 20 min under ambient conditions. The substrates were then transferred into a nitrogen-filled glovebox. The active layer was spin-coated on the PEDOT:PSS layer from 11 mg/ml chloroform solution (D:A = 1:1.6) at 4000 rpm for 30 s with 0.25 vol% DIO as additives. After spin-coating, the active layer was annealed at 90 °C for 5 min and then 1.0 mg/ml PDINN methanol solution was deposited on the active layer. The device fabrication was completed by thermally evaporating 80 nm thick silver under vacuum at a pressure of 1 × 10^-5^ Pa. The active area of the device was *ca.* 4 mm^2^.

**Device Characterization**

The current density–voltage (*J–V*) characteristics of unencapsulated photovoltaic devices were measured under N_2_ using a Keithley 2400 source meter. A 300 W xenon arc solar simulator (Oriel) with an AM 1.5 global filter operated at 100 mW cm^−2^ was used to simulate the AM 1.5G solar irradiation. The illumination intensity was corrected by using a silicon photodiode with a protective KG5 filter calibrated by the National Renewable Energy Laboratory (NREL). The devices were tested at room temperature (ca. 25 ℃) in glove box under N_2_ atmosphere. The scan voltage from -1.5 to 1.5 V with a voltage step of 10 mV and a dwell time of 1 ms. The EQE spectra were obtained using a QE-R Solar Cell EQE measurement system (Model:XES-70S1, Enlitech) using a lock-in amplifier with a monochromator and 300 W xenon lamp. A calibrated silicon detector (RC-S10A, Enlitech) was utilized as a reference.

**Calculation of wetting coefficient (ω)**

The interfacial interaction energy (γ_A1/A2_) can be calculated according to the Neumann equation:

$$\gamma_{A_{1}/A_{2}}=\gamma_{A_{1}}+\gamma_{A_{2}}-2\sqrt{\gamma_{A_{1}}\gamma_{A_{2}}}e^{\left[ {-\beta\left( \gamma_{A_{1}}-\gamma_{A_{2}} \right)}^{2} \right]}$$

where *β* is 0.000115 m^4^ mJ^-2^.

Therefore, the ω_A2_ can be calculated following the equation of Young’s equation:

$$\omega_{A_{2}}=\frac{\gamma_{A_{1}/A_{2}}-\gamma_{D/A_{2}}}{\gamma_{D/A_{2}}}$$

If ω_A2_>1, component A_2_ will lie in the domain of component D; if ωA_2_ < −1, component A_2_ will lie in the domain of component A_1_; if −1 < ωA_2_ < 1, component A_2_ will be located at the interfaces of D and A_1_. The D, A_1_ and A_2_ represent host donor, host acceptor and guest acceptor, respectively.

**Energy loss analysis**

The 𝐸_𝑙𝑜𝑠𝑠_ in organic solar cells can be divided into three parts:

$$E_{loss}=E_{g}-qV_{OC}$$

$$=\left( E_{g}-qV_{OC}^{SQ} \right) + \left( qV_{OC}^{SQ}-qV_{OC}^{rad} \right) +\left( qV_{OC}^{rad}-qV_{OC} \right)$$

$=\Delta E_{1}+\Delta E_{2}+\Delta E_{3}$

where *E*_g_ is the bandgap and can be expressed by equation of $E_{g}=\frac{\int_{a}^{b} E_{g}\cdot p\left( E_{g} \right)dE_{g}}{\int_{a}^{b} p(E_{g})dE_{g}}$, The integral boundaries a and b are selected where P(a) = P(b) = 0.5max[P(E_g_)].

$V_{OC}^{SQ}$ in the equation is the maximum voltage based on the Shockley-Queisser limit, which can be calculated by the equation of $V_{OC}^{SQ}= \frac{kT}{q}\ln\left( \frac{J_{SC}^{SQ}}{J_{0}^{SQ}}+1 \right)\cong\frac{kT}{q}\ln\left( \frac{q\cdot\int_{E_{g}}^{+\infty} \emptyset_{AM1.5G}\left( E \right)\cdot dE}{q\cdot\int_{E_{g}}^{+\infty} \emptyset_{BB}\left( E \right)\cdot dE} \right)$. The ∆*E*_1_ equals to $E_{g}-qV_{OC}^{SQ}$.

In generally, ∆*E*_3_ can be calculated from the electroluminescence quantum efficiency (*EQE*_EL_) measurement, in which ∆*E*_3_ equals to $-kT({EQE}_{EL})$.

Therefore, the ∆*E*_2_ can be calculated from the equation of $E_{loss}-\Delta E_{1}-\Delta E_{3}$.

**SCLC Mobility Measurements**

Space charge-limited currents were tested in electron-only devices with a configuration of ITO/ZnO/active layer/PDINN/Ag and hole-only devices with a configuration of ITO/PEDOT:PSS/active layer/Au. The devices were prepared following the same procedure described in the experimental section for photovoltaic devices, except for the metal electrode. The mobilities were determined by fitting the dark current to the model of a single carrier SCLC current with field dependent mobility, which is described as

$J=\frac{9\varepsilon_{0}\varepsilon_{r}\mu_{0}V^{2}}{8L^{3}}$

Where *J* is the current, *μ*_0_ is the zero-field mobility, ε_0_ is the permittivity of free space, ε_r_ is the relative permittivity of the material, *V* is the effective voltage, and *L* is the thickness of the active layer.

**Atomic Force Microscopy (AFM)**

Atomic force microscopy (AFM) images of the thin films were obtained on a NanoscopeIIIa AFM (Digital Instruments) operating platform in tapping mode. The samples were prepared by spinning coated the active layers on the PEDOT:PSS layer. The corresponding blend samples were thermally annealed for 10 mins.

**Transmission electron microscopy (TEM)**

Transmission electron microscopy (TEM) observation was performed on JEOL 2200 FS at 160 kV accelerating voltage. The samples for electron microscopy were prepared by dissolving the PEDOT:PSS layer using water and transferring the floating active layer to the TEM grids.

**Grazing incidence wide-angle X-ray scattering (GIWAXS)**

The grazing incidence wide-angle X-ray scattering (GIWAXS) characterization of the thin films was performed at the Advanced Light Source (ALS) on the beamline of 7.3.3. Samples were prepared under device conditions on the Si/PEDOT:PSS substrates.

**The synthesis of BTP-γ-Br and BTP-δ-Br**

**Scheme 1.** The synthetic route for BTP-γ-Br and BTP-δ-Br.

**Compound BTP-γ-Br**

Compound TPBT-CHO (155 mg, 0.15 mmol, 1 eq), γ-Br (164 mg, 0.6 mol, 4 eq) and pyridine (0.1 ml) was added in chloroform (15 ml). The mixture was stirred at 70 °C overnight. After cooling to room temperature, the solvent was removed in vacuum and the residue was purified by column chromatography to give a dark blue solid (186 mg, 81%). ^1^H NMR (400 MHz, CDCl_3_):δ 9.20 (s, 2H), 8.58 (d, *J* = 8.4 Hz, 2H), 8.06 (s, 2H), 7.88 (d, *J* = 8.5 Hz, 2H), 4.77 (d, *J* = 7.0 Hz, 4H), 3.24 (d, *J* = 7.3 Hz, 4H), 2.09 (s, 2H), 1.89 (s, 4H), 1.45 – 0.46 (m, 67H). ^13^C NMR (176 MHz, CDCl_3_) δ 187.10, 160.04, 153.70, 147.55, 145.10, 138.56, 138.35, 137.78, 137.70, 135.82, 135.65, 133.83, 133.43, 130.48, 129.93, 129.90, 129.47, 126.79, 126.46, 120.23, 115.27, 114.91, 113.56, 77.20, 77.01, 76.83, 68.37, 55.62, 40.35, 39.22, 38.88, 38.83, 38.67, 37.11, 36.65, 36.35, 35.92, 34.34, 34.27, 32.48, 31.91, 31.71, 31.57, 31.47, 31.24, 30.10, 29.90, 29.85, 29.80, 29.78, 29.70, 29.63, 29.55, 29.52, 29.48, 29.37, 29.32, 29.24, 29.13, 28.87, 27.98, 27.95, 27.86, 27.66, 27.62, 27.40, 27.25, 27.21, 25.54, 23.23, 23.21, 22.78, 22.69, 22.65, 22.63, 22.55, 20.13, 19.53, 19.17, 19.13, 14.12, 14.09, 13.72, 11.39, 10.22, 10.18. HRMS (MALDI-TOF) calcd for C_82_H_88_Br_2_N_8_O_2_S_5_ [M]+:1537. 76, found, 1534.40.


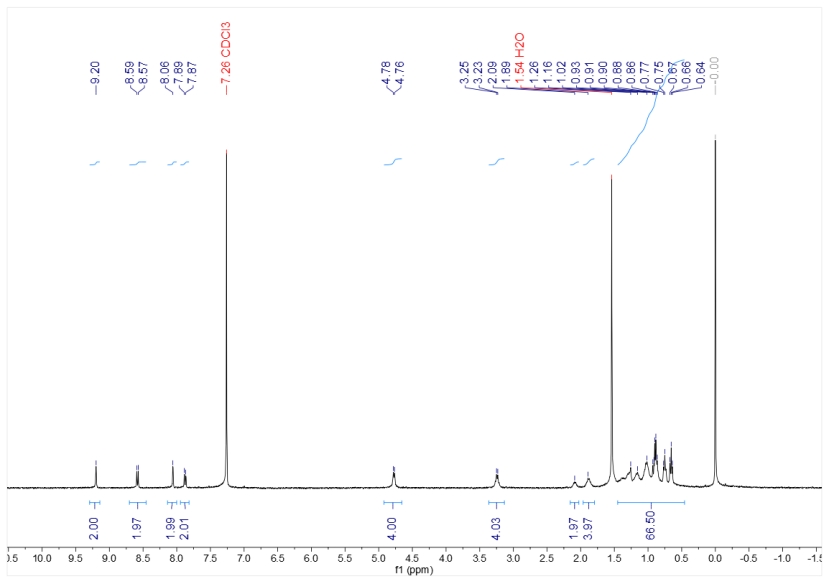


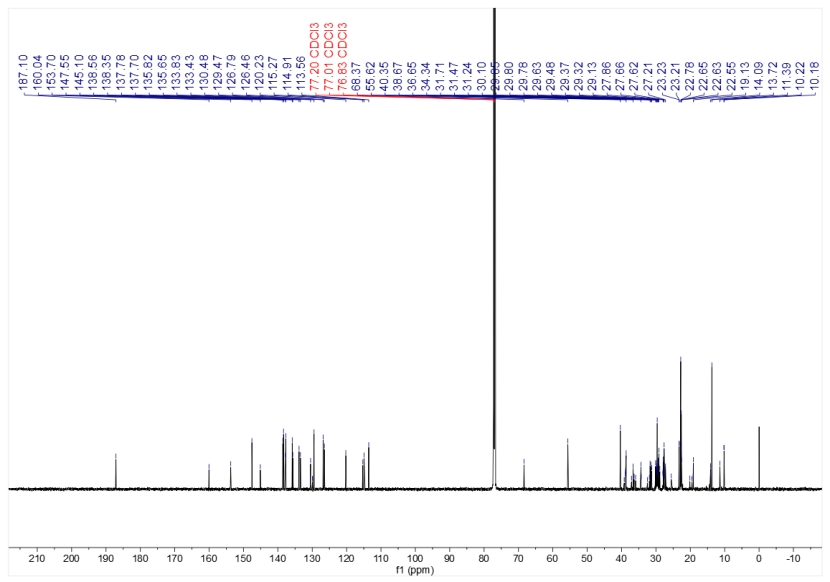


**Compound BTP-δ-Br**

Compound TPBT-CHO (155 mg, 0.15 mmol, 1 eq), δ-Br (164 mg, 0.6 mol, 4 eq) and pyridine (0.1 ml) was added in chloroform (15 ml). The mixture was stirred at 70 °C overnight. After cooling to room temperature, the solvent was removed in vacuum and the residue was purified by column chromatography to give a dark blue solid (106 mg, 46%). ^1^H NMR (400 MHz, CDCl_3_):δ 9.19 (s, 2H), 8.86 (s, 2H), 7.88 (d, *J* = 8.4 Hz, 2H), 7.80 (d, *J* = 7.8 Hz, 2H), 4.77 (s, 4H), 3.24 (d, *J* = 6.9 Hz, 4H), 2.09 (s, 2H), 1.89 (s, 4H), 1.45 – 0.54 (m, 59H). ^13^C NMR (176 MHz, CDCl_3_) δ 187.52, 159.46, 153.70, 147.56, 145.11, 141.42, 137.83, 137.27, 135.88, 135.58, 135.49, 133.78, 133.46, 130.44, 130.20, 129.90, 128.22, 124.60, 120.30, 115.13, 114.58, 113.59, 77.19, 77.01, 76.83, 68.90, 55.62, 40.33, 38.83, 38.67, 36.65, 35.91, 34.34, 31.91, 31.71, 31.46, 31.23, 30.10, 29.90, 29.77, 29.70, 29.64, 29.55, 29.52, 29.48, 29.36, 29.32, 29.24, 29.12, 28.86, 27.94, 27.86, 27.66, 27.21, 25.53, 23.21, 23.18, 22.80, 22.68, 22.62, 22.55, 19.17, 19.13, 14.12, 14.09, 13.72, 11.39, 10.21, 10.15. MS (MALDI-TOF) calcd for C_82_H_88_Br_2_N_8_O_2_S_5_ [M]+:1537. 76, found, 1537.40.


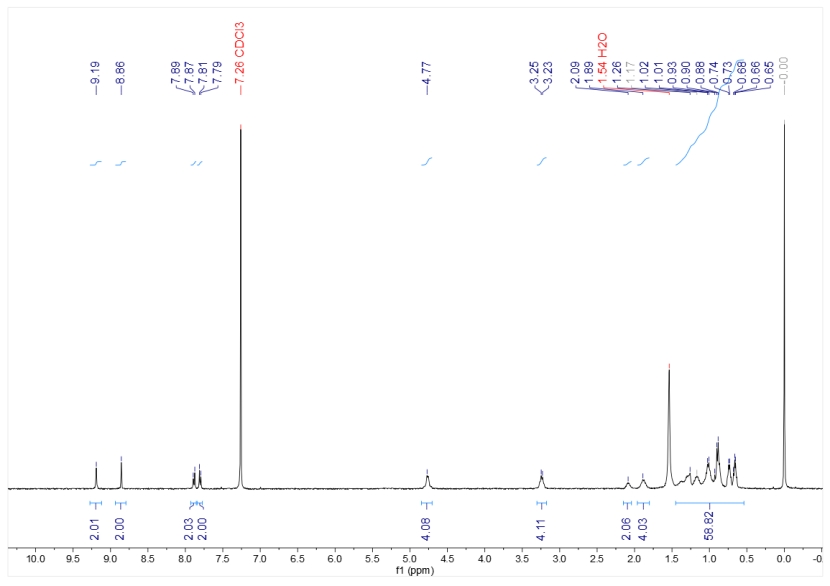


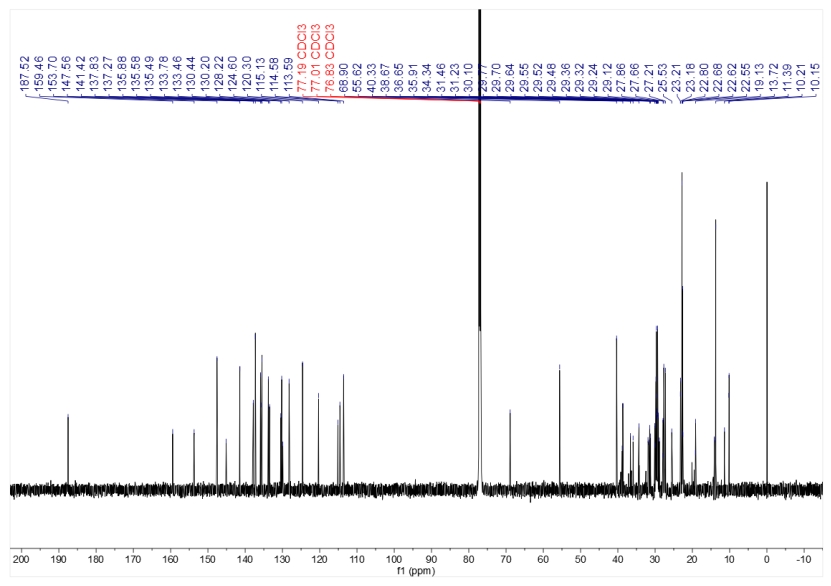

**Figure S1.** Thermal gravimetric analysis of BTP-γ-Br and BTP-δ-Br.


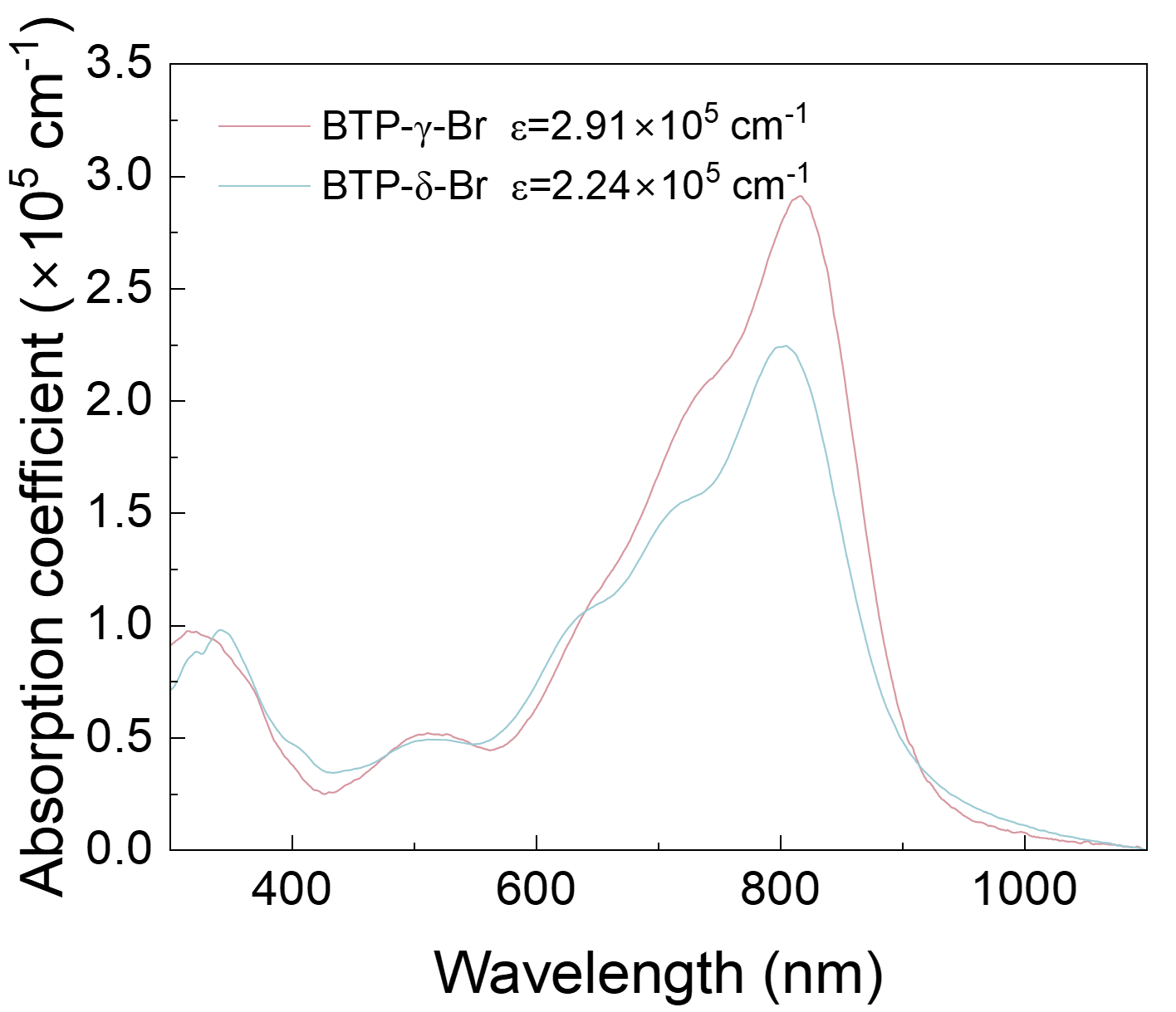


**Figure S2.** The absorption coefficient of BTP-γ-Br and BTP-δ-Br neat films.


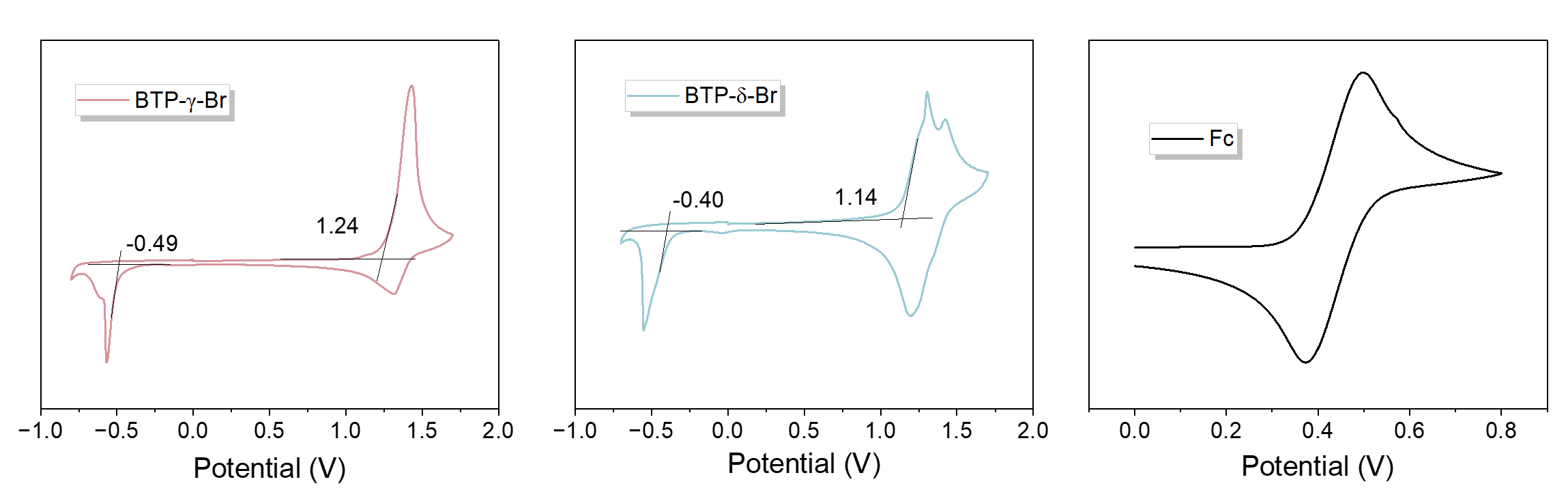


**Figure S3.** Cyclic voltammograms (CV) of BTP-γ-Br and BTP-δ-Br thin films in 0.1 M *n*-Bu4PF6 acetonitrile solution, in which ferrocene as reference compound.


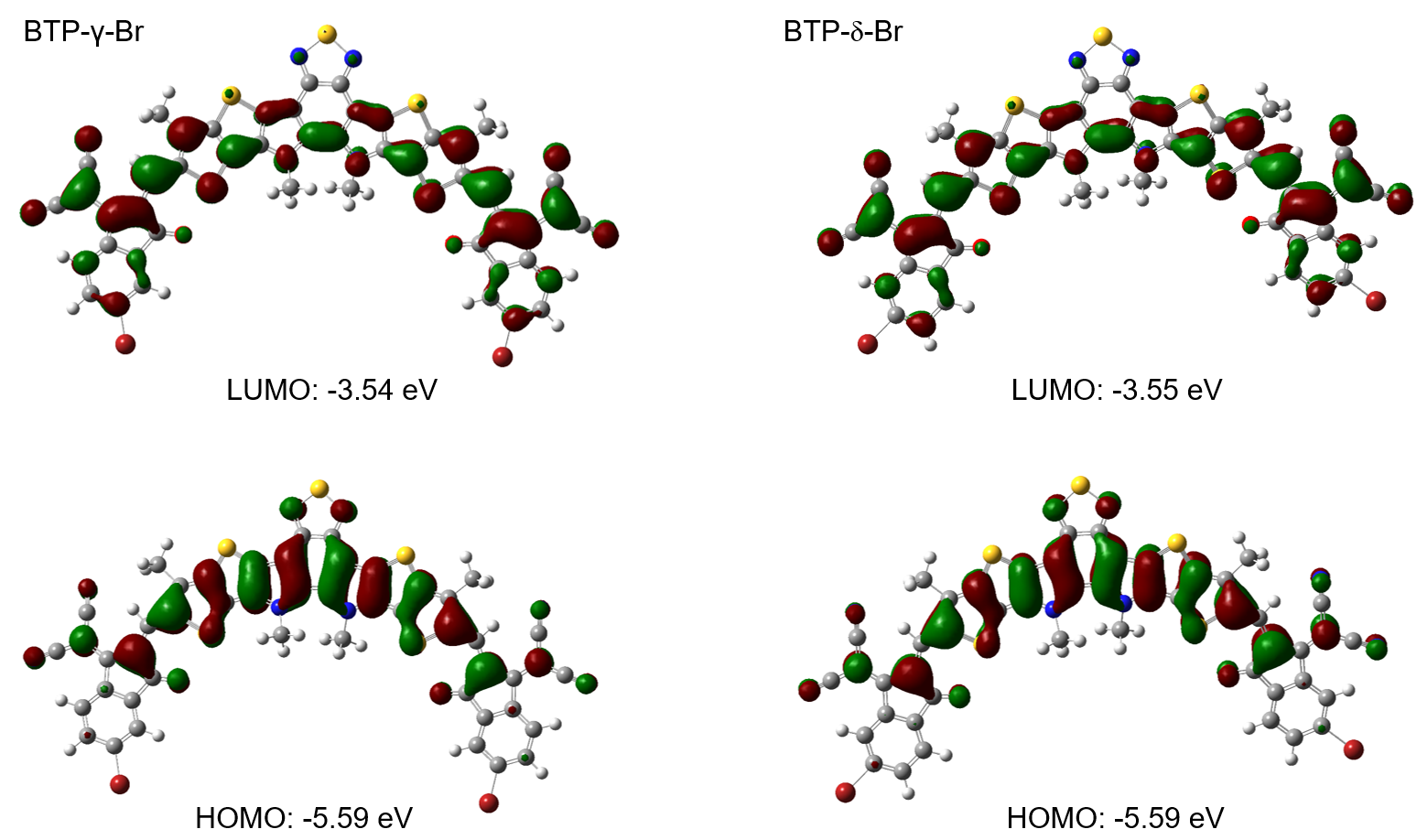


**Figure S4.** The DFT calculations for the frontier molecular orbits of BTP-γ-Br and BTP-δ-Br.


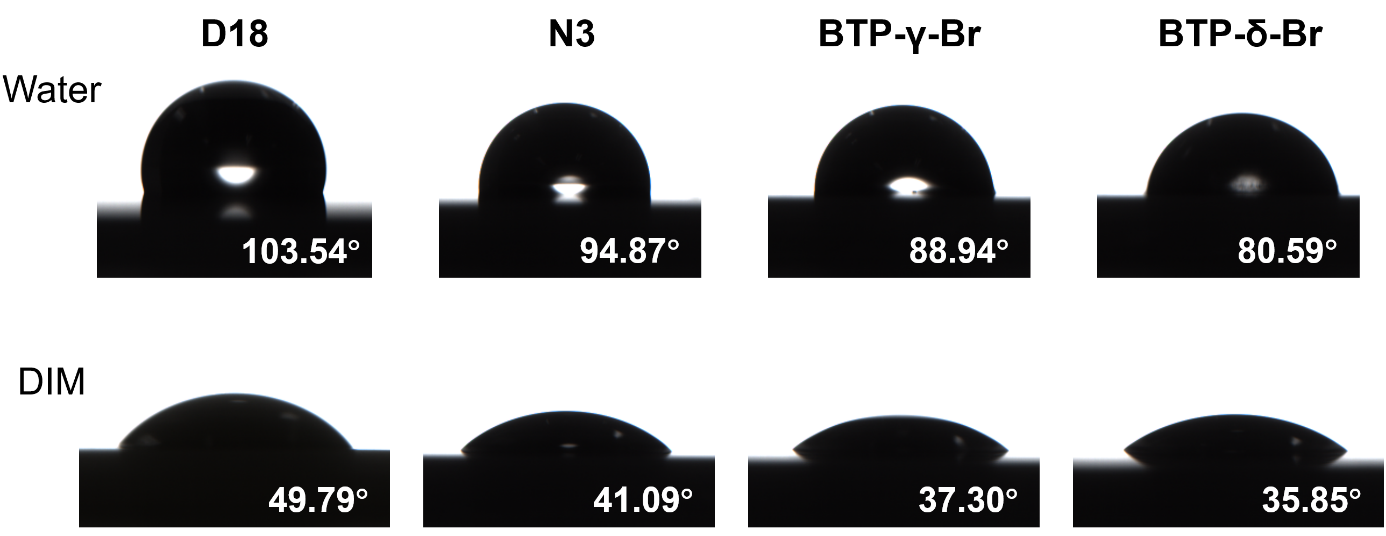


**Figure S5.** The contact angle of D18, N3, BTP-γ-Br and BTP-δ-Br. The DIM represents diiodomethane.


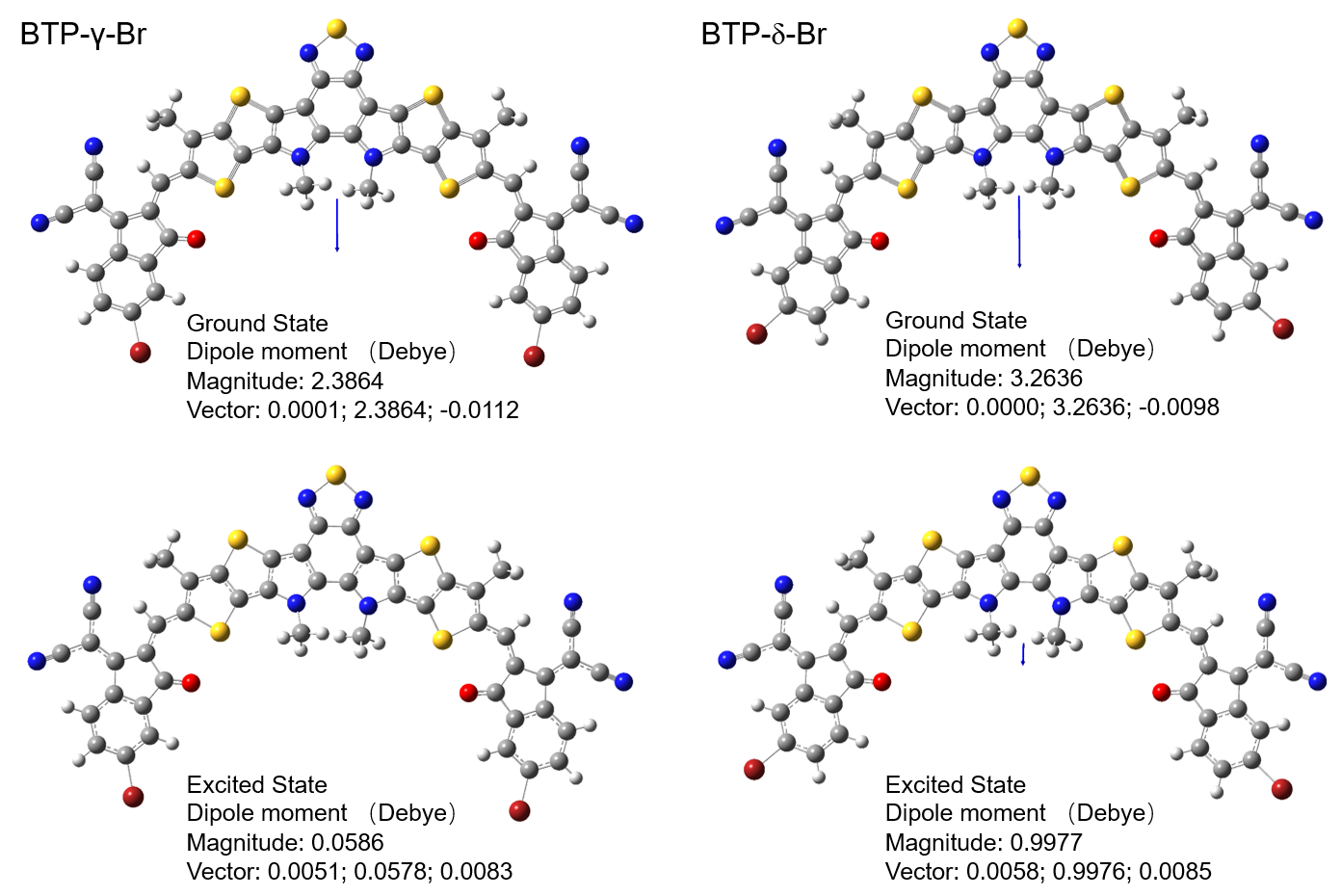


**Figure S6.** The DFT calculations of BTP-γ-Br and BTP-δ-Br at group state and excited state, where the dipole moment is drawn by the blue arrow.


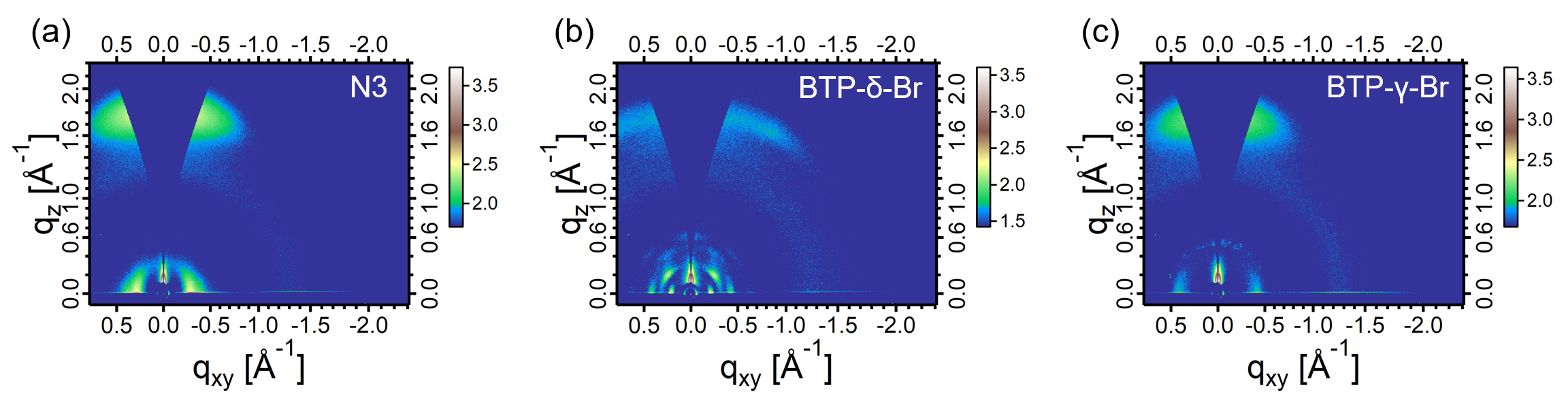


**Figure S7.** The 2D GIWAXS patterns of the (a) N3, (b) BTP-δ-Br and (c) BTP-γ-Br neat films.


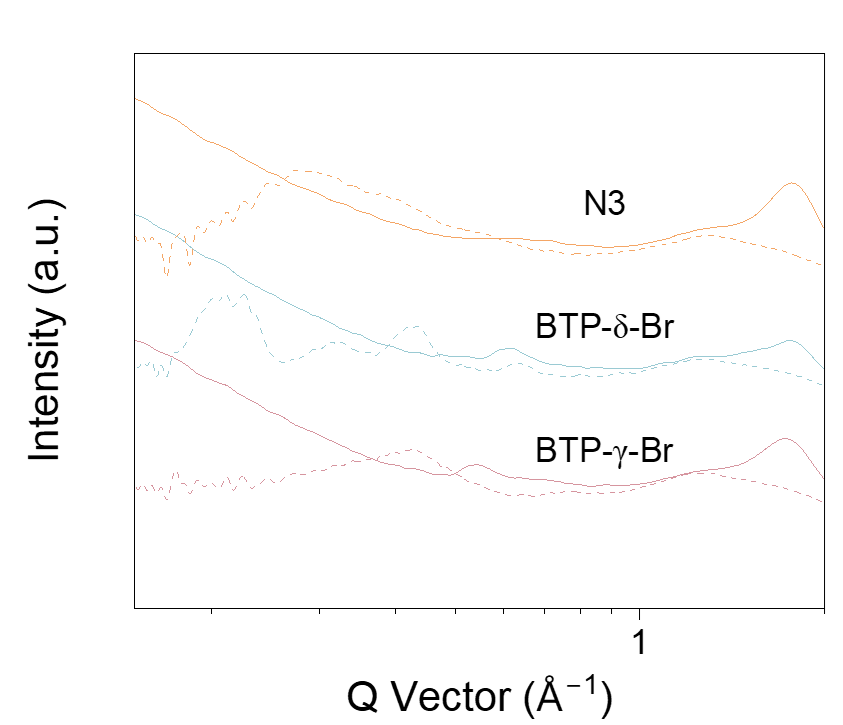


**Figure S8.** The 1D GIWAXS results of the N3, BTP-δ-Br and BTP-γ-Br neat films. The dashed line represents the in-plane (IP) direction and the solid line represents the out-of-plane (OOP) direction.


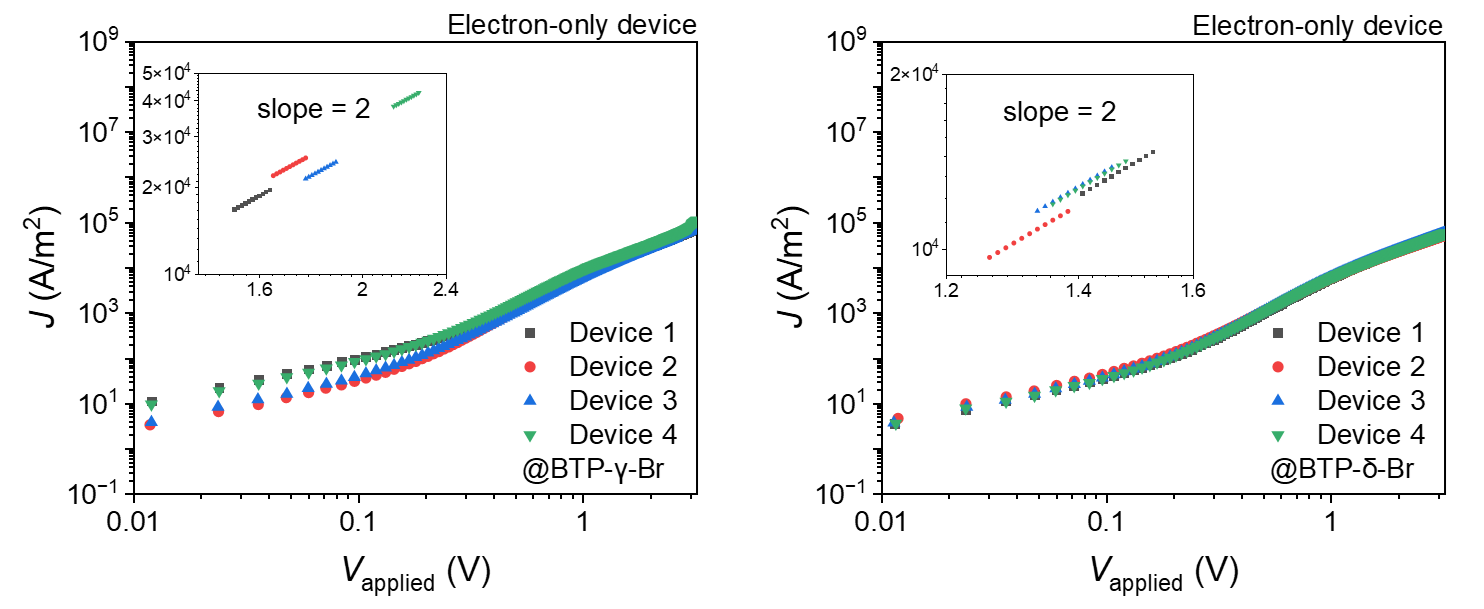


**Figure S9.** Space-charge limited current (SCLC) characteristics of electron-only devices based on acceptor neat films.


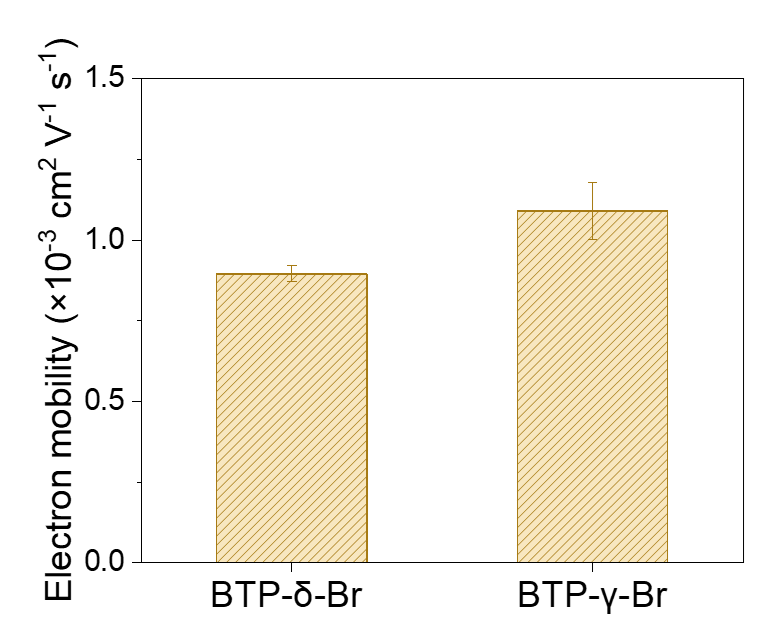


**Figure S10.** The electron mobilities of BTP-γ-Br and BTP-δ-Br thin films, where the error bars represent the standard error of the mean across four devices.


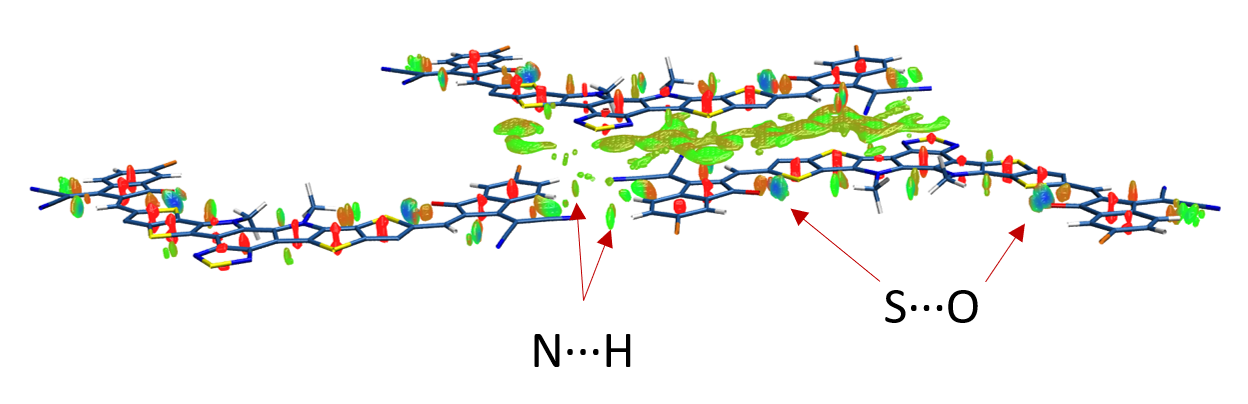


**Figure S11.** The NCI analysis on the trimer extracted from the single crystal structure of BTP-δ-Br.


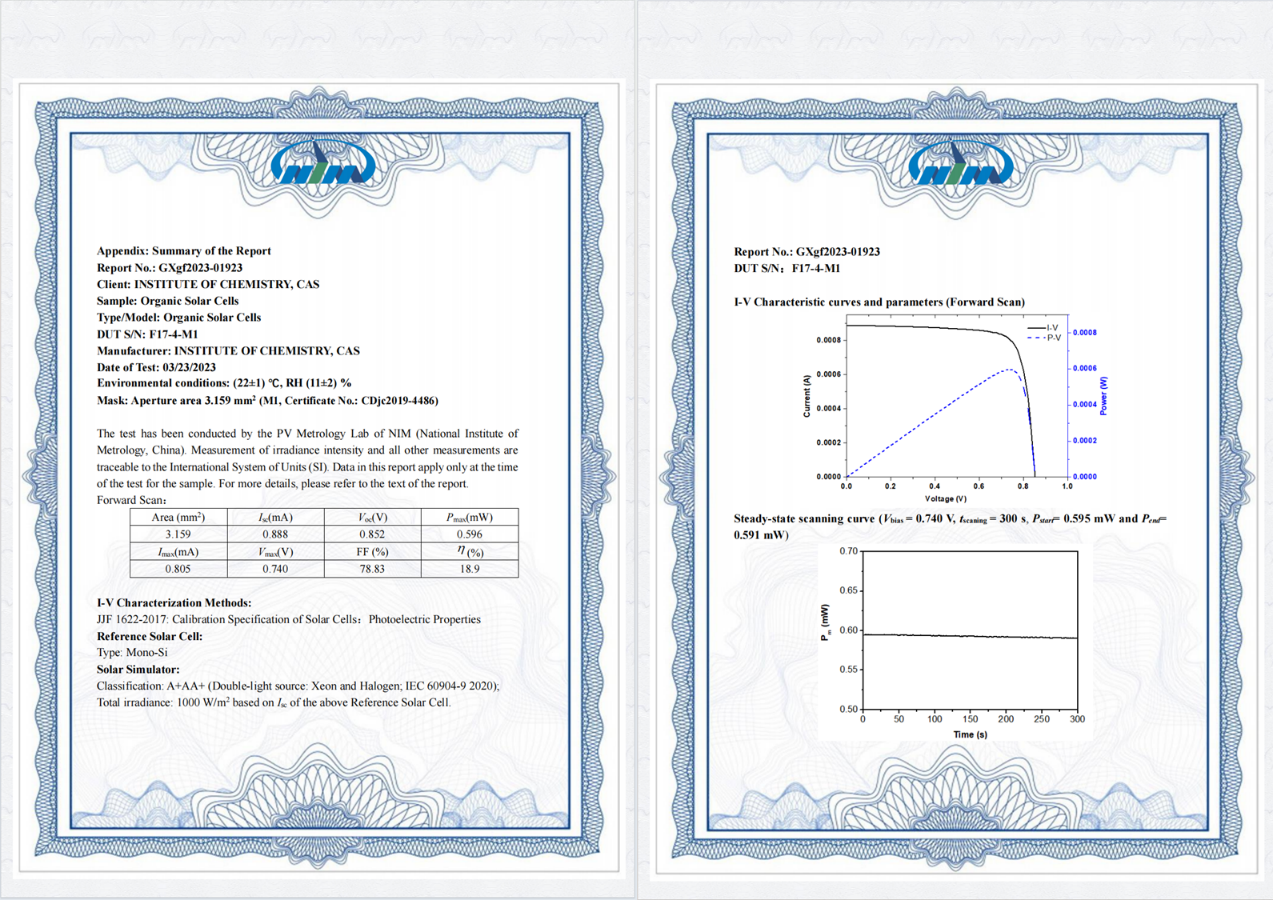


**Figure S12.** The certified photovoltaic performance of the optimized ternary solar cells from the National Institute of Metrology, China (NIM).


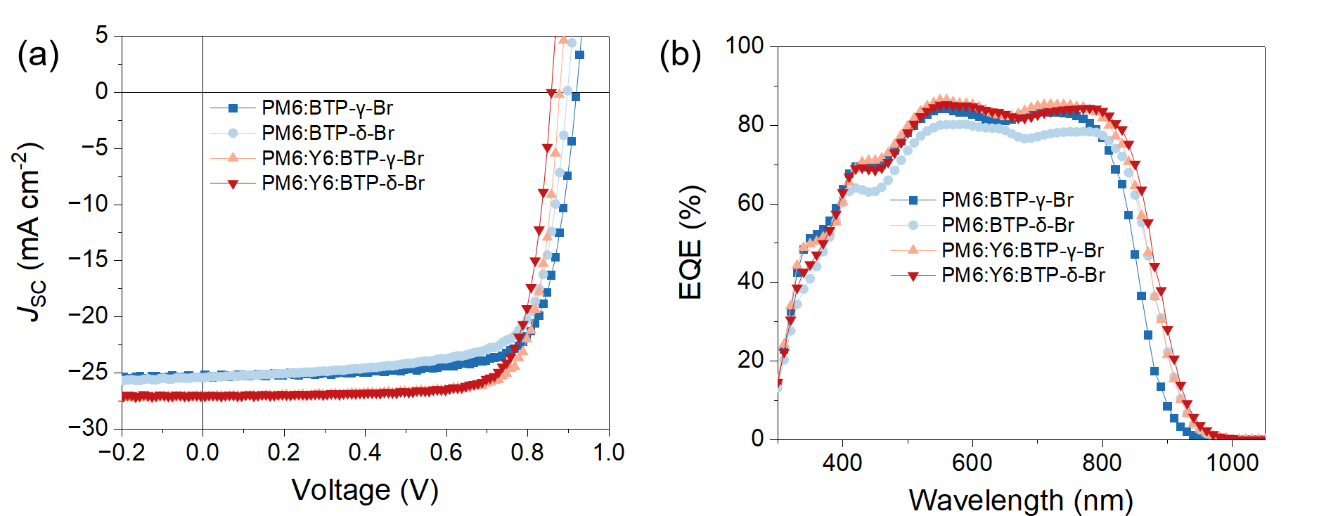


**Figure S13.** (a)The J-V curves and (b) EQE spectra for binary and ternary devices.


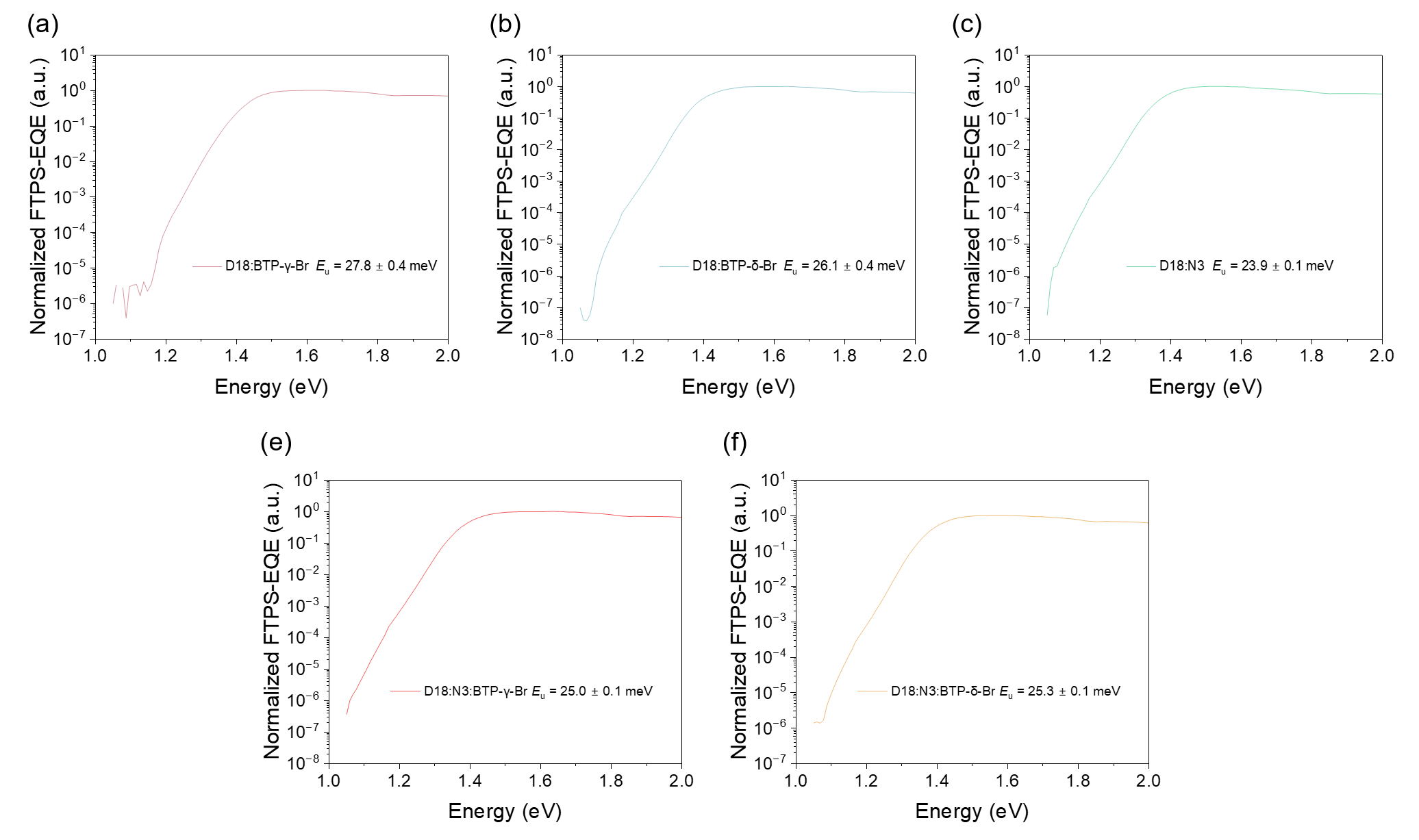


**Figure S14.** (a-f) FTPS-EQE of binary and ternary devices. *E*_u_ is Urbach energy, which is obtained from the FTPS-EQE curves with the exponential fitting ($\alpha(E)=\alpha_{0}\cdot exp(\frac{E-E_{g}}{E_{u}})$).


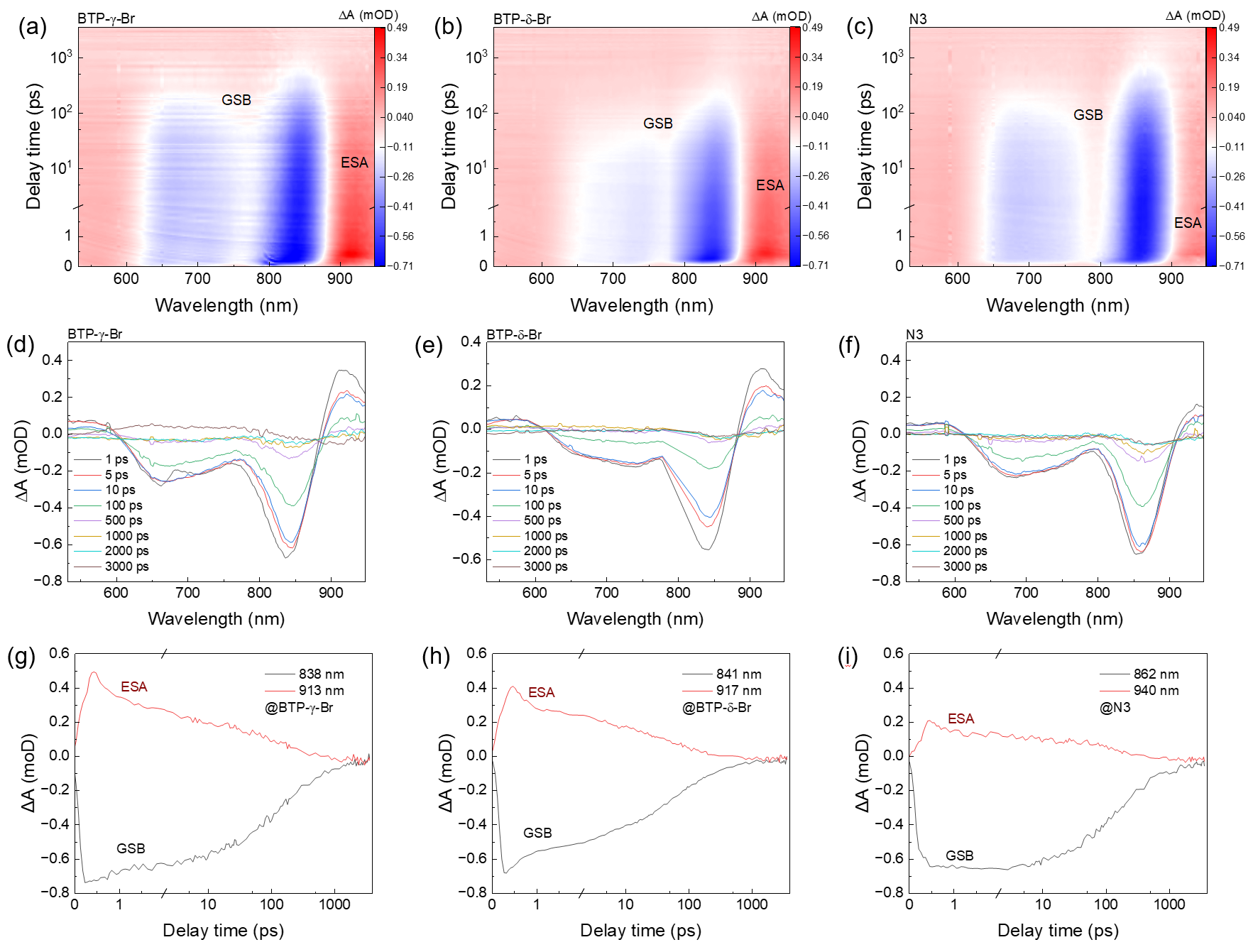


**Figure S15.** The 2D transient absorption (TA) spectra of BTP-γ-Br, BTP-δ-Br and N3 neat films (a,b,c), the corresponding 1D profiles at different delay time (d,e,f) and the dynamics of ground state bleaching (GSB) and excited sate absorption (ESA) signals (g,h,i).


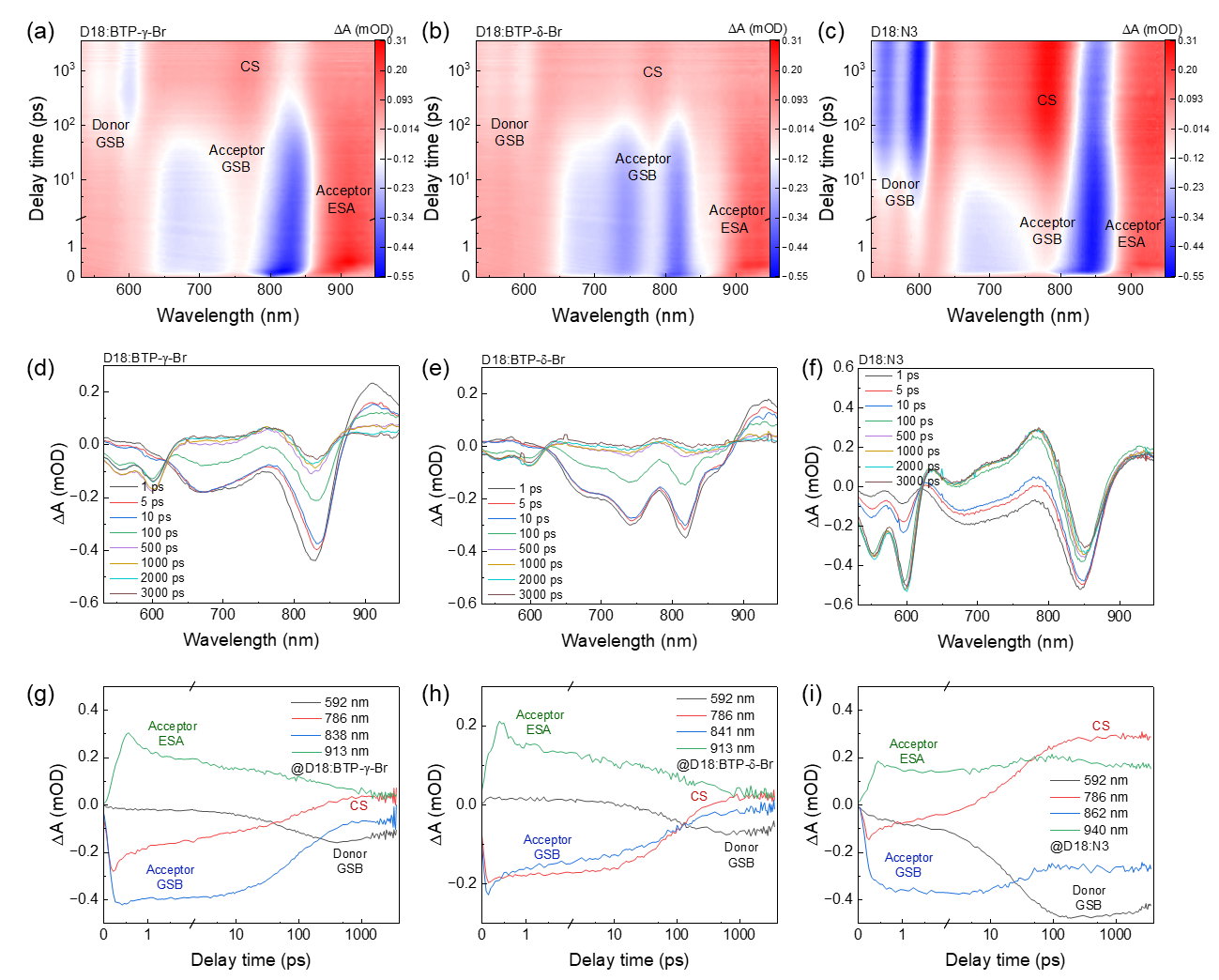


**Figure S16.** The 2D transient absorption (TA) spectra of D18:BTP-γ-Br, D18:BTP-δ-Br, D18:N3 thin films (a,b,c), the corresponding 1D profiles at different delay time (d,e,f) and the dynamics of ground state bleaching (GSB), excited sate absorption (ESA) and charge separation (CS) signals (g,h,i).


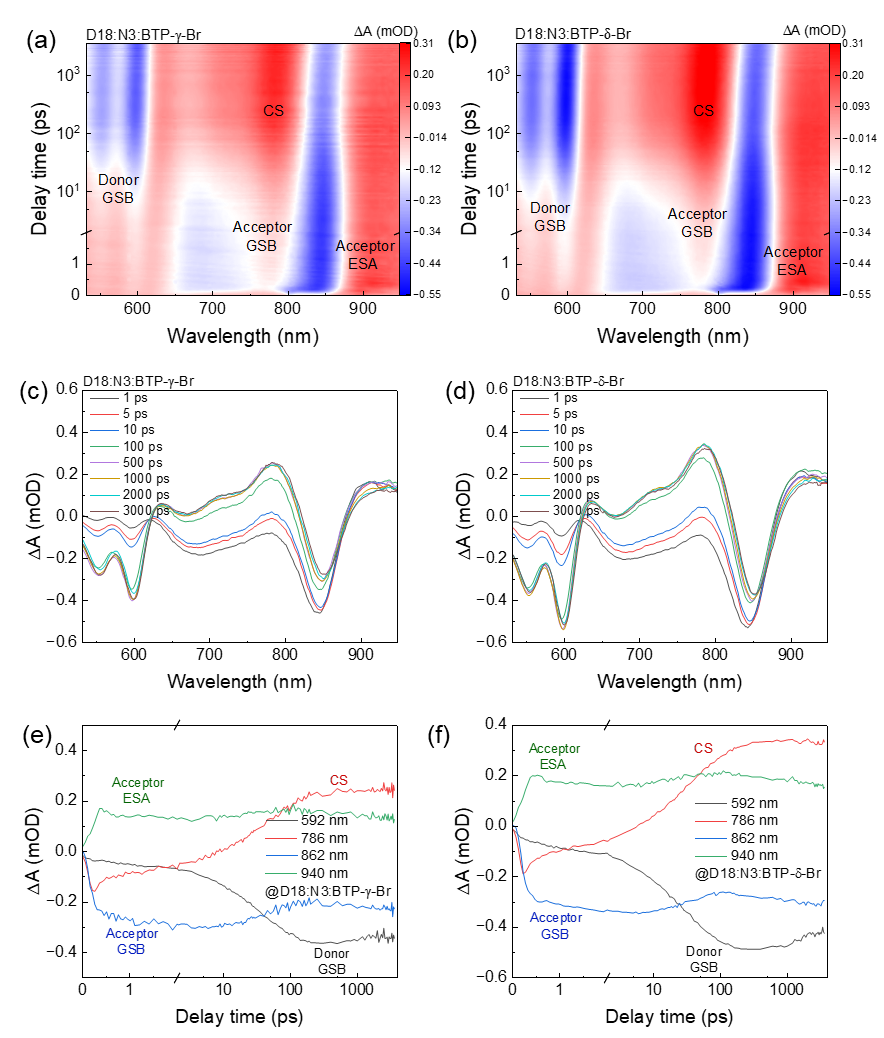


**Figure S17.** The 2D transient absorption (TA) spectra of D18:BTP-γ-Br:N3 and D18:BTP-δ-Br:N3 ternary thin films (a,b), the corresponding 1D profiles at different delay time (c,d) and the dynamics of ground state bleaching (GSB), excited sate absorption (ESA) and charge separation (CS) signals (e,f).


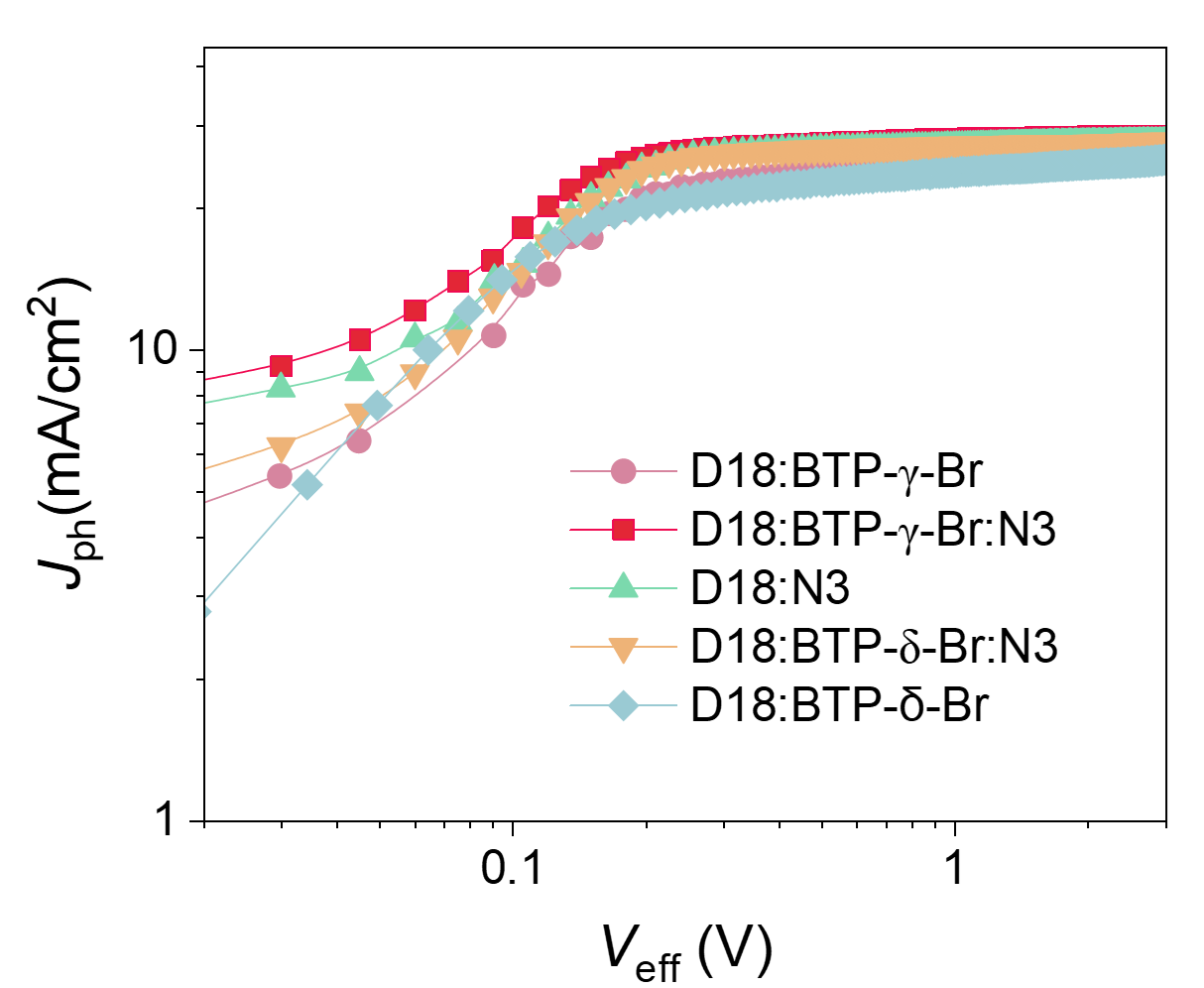


**Figure S18.** The *J*_ph_ versus *V*_eff_ curves of the optimal devices.


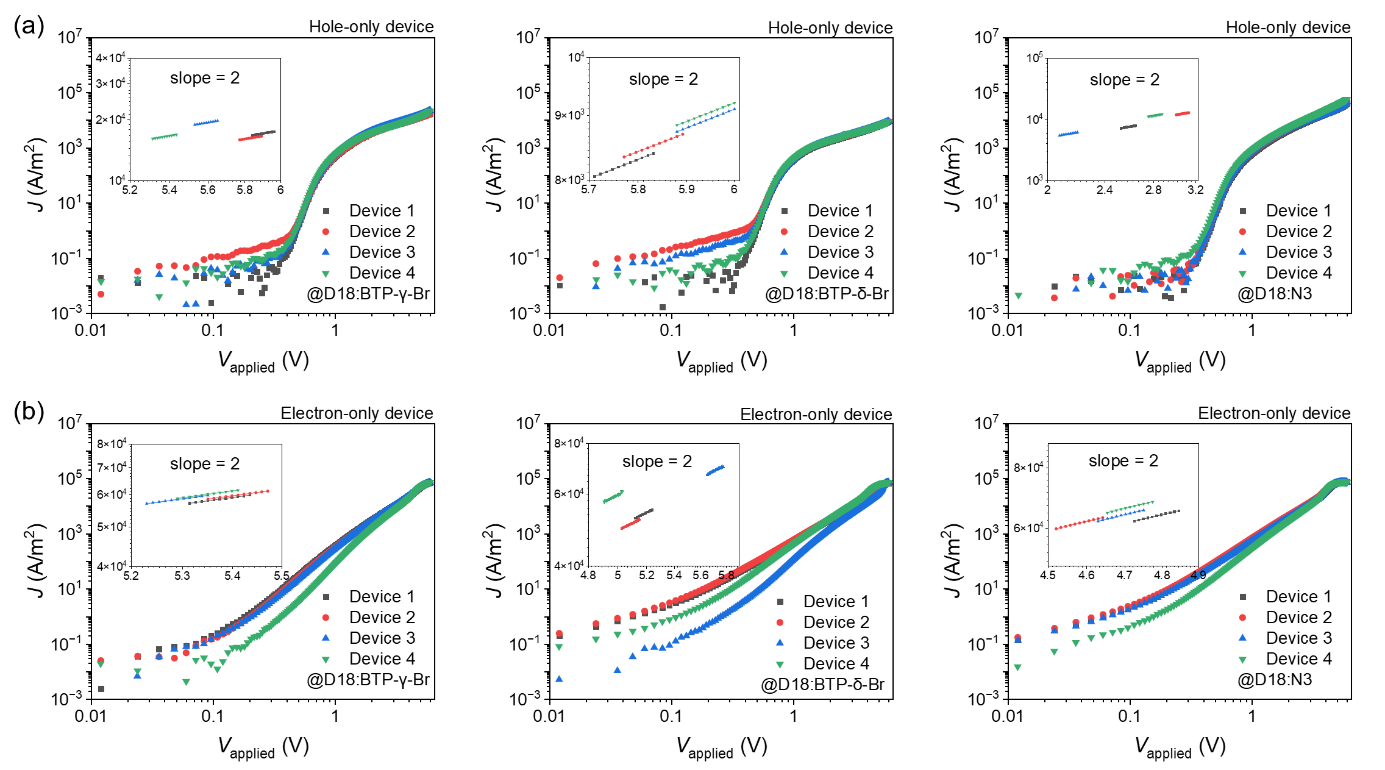


**Figure S19.** Space-charge limited current (SCLC) characteristics of (a) hole-only devices and (b) electron-only devices based on binary films.


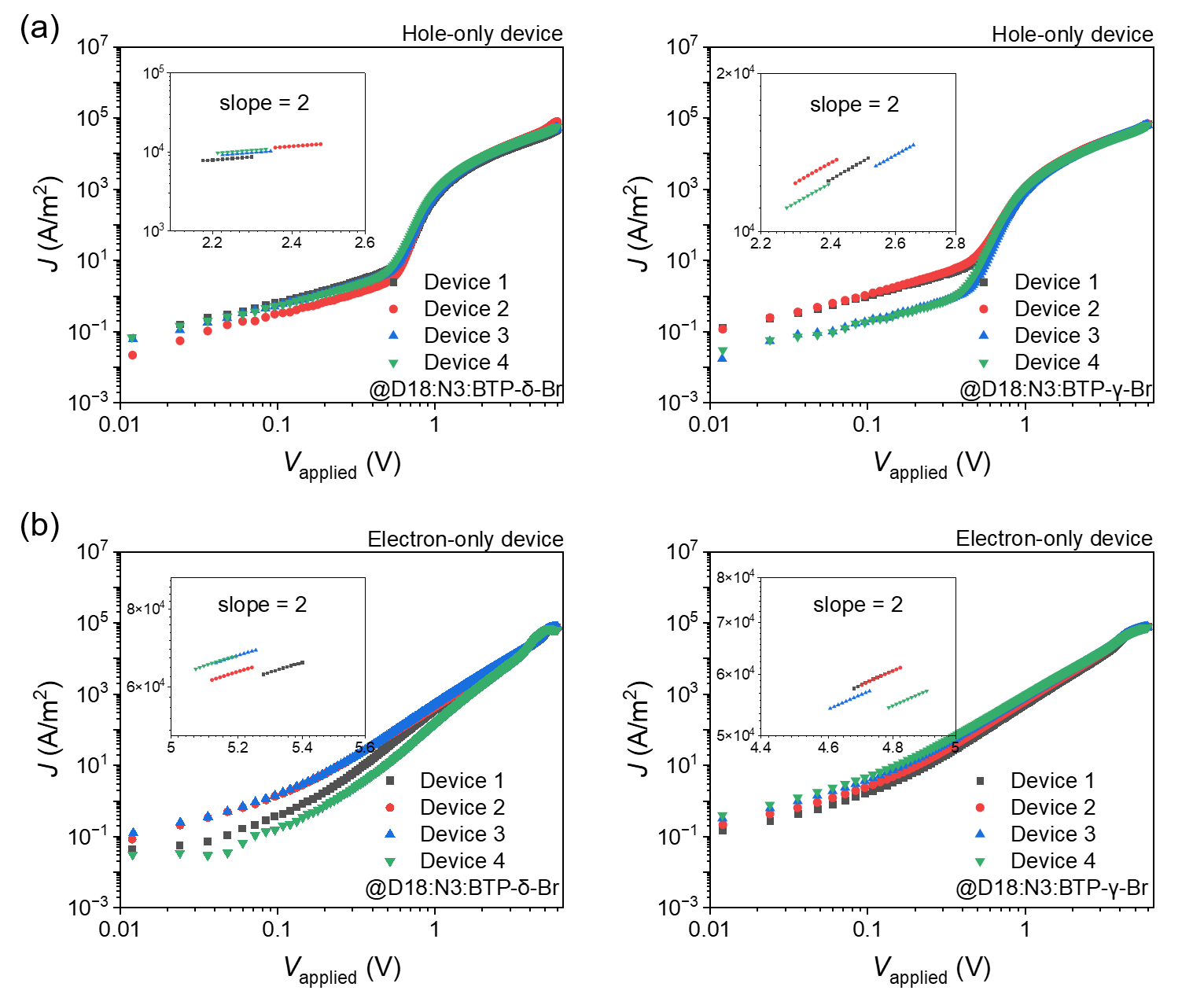


**Figure S20.** Space-charge limited current (SCLC) characteristics of (a) hole-only devices and (b) electron-only devices based on ternary films.


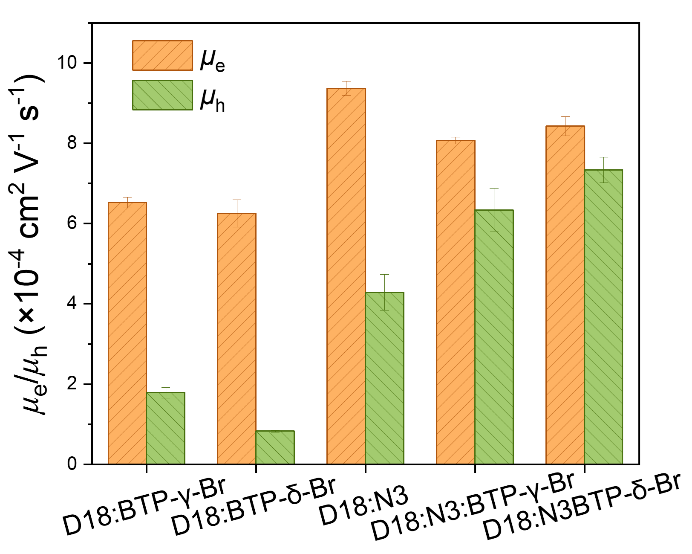


**Figure S21.** The hole mobilities (*μ*_h_) and electron mobilities (*μ*_e_) of binary and ternary devices, where the error bars represent the standard error of the mean across four devices.

**Table S1.** The surface energy characteristics of the D18, N3, BTP-γ-Br and BTP-δ-Br pristine films.

| **Materials** | ***θ*_water_**  [ deg ] | ***θ*_DIM_**  [ deg ] | **γ**  [mN m^-1^] | ***χ*_D-A_** | ***χ*_A1-A2_** | ω |
| --- | --- | --- | --- | --- | --- | --- |
| D18 | 103.54 | 49.79 | 35.70 | - | - | - |
| N3 | 94.87 | 41.09 | 39.46 | 0.018K | - | - |
| BTP-γ-Br | 88.94 | 39.30 | 40.97 | 0.063K | 0.007K | -1.805 |
| BTP-δ-Br | 80.59 | 35.85 | 42.06 | 0.113K | 0.041K | -2.386 |

**Table S2.** The detailed GIWAXS data in the out-of-plane direction of neat films.

| **Materials** | **(010) peaks** | | | |
| --- | --- | --- | --- | --- |
|  | ***q***  (Å^-1^) | **d-spacing**  (Å) | **FWHM**  (Å^-1^) | **CCL**  (Å) |
| N3 | 1.683 | 3.73 | 0.222 | 25.4 |
| BTP-δ-Br | 1.679 | 3.74 | 0.204 | 27.7 |
| BTP-γ-Br | 1.685 | 3.73 | 0.219 | 25.8 |

**Table S3.** The detailed GIWAXS data in the in-plane direction of neat films.

| **Materials** | **(100) peaks** | | | |
| --- | --- | --- | --- | --- |
|  | ***q***  (Å^-1^) | **d-spacing**  (Å) | **FWHM**  (Å^-1^) | **CCL**  (Å) |
| N3 | 0.309 | 20.3 | 0.0620 | 91.2 |
| BTP-δ-Br | 0.422 | 14.9 | 0.0750 | 75.4 |
| BTP-γ-Br | 0.312 | 20.1 | 0.0616 | 91.8 |

**Table S4.** The calculated quadrupole moment for BTP-δ-Br and BTP-γ-Br.

| Materials | *Q*_XX_  (Debye) | *Q*_YY_  (Debye) | *Q*_ZZ_  (Debye) |
| --- | --- | --- | --- |
| BTP-γ-Br | -142.2 | 67.9 | 74.2 |
| BTP-δ-Br | -181.6 | 94.3 | 87.3 |

**Table S5.** The crystallographic data of BTP-γ-Br.

| **Identification code** | **BTP-γ-Br** |
| --- | --- |
| l | C_84_H_92_Br_2_N_8_O_2_S_5_ |
| Formula weight | 1537.77 |
| Temperature/K | 170 |
| Crystal system | monoclinic |
| Space group | I2/a |
| a/Å | 14.8169(2) |
| b/Å | 18.8331(3) |
| c/Å | 26.7853(3) |
| α/° | 90 |
| β/° | 94.493(1) |
| γ/° | 90 |
| Volume/ Å^3^ | 7451.42(18) |
| Z | 4 |
| *ρ*_calc_g/cm^3^ | 1.330 |
| *μ*/mm^-1^ | 3.105 |
| F(000) | 3086.0 |
| Crystal size/mm^3^ | 0.32 × 0.28 × 0.25 |
| Radiation | Cu Kα (λ = 1.54184) |
| 2Θ range for data collection/° | 6.620 to 153.872 |
| Reflections collected | 7482 |
| Independent reflections | 5965 [R_int_ = 0.0230, R_sigma_ = 0.0172] |
| Data/restraints/parameters | 7482/332/541 |
| Goodness-of-fit on F^2^ | 2.313 |
| Final R indexes [I>=2σ (I)] | R_1_ = 0. 0915, wR_2_ = 0.2819 |
| Final R indexes [all data] | R_1_ = 0.1038, wR_2_ = 0.2936 |
| Largest diff. peak/hole / e Å^-3^ | 1.906/-1.015 |

**Table S6.** The detailed photovoltaic parameters of binary and ternary devices.

| **Blends**^a^ | ***V*_OC_**  (V) | ***J*_SC_**  (mA cm^-2^) | **FF**  (%) | **PCE**  (%) |
| --- | --- | --- | --- | --- |
| PM6:BTP-γ-Br | 0.920 | 25.3 | 75.4 | 17.6 |
| PM6:BTP-δ-Br | 0.897 | 25.4 | 73.0 | 16.6 |
| PM6:Y6: BTP-γ-Br | 0.878 | 27.1 | 78.8 | 18.8 |
| PM6:Y6:BTP-δ-Br | 0.858 | 27.0 | 78.4 | 18.2 |

^a^The D:A ratio is 1:1.2 for binary devices and 1:0.9:0.3 for ternary devices.

**Table S7.** The detailed energy loss data of binary and ternary devices.

| **Devices** | ***E_g_***^a^  [eV] | ***V*_OC_**  [V] | ***E*_loss_**  [eV] | ***V*_OC,SQ_**  [V] | **∆*E*_1_**  [eV] | **∆*E*_2_**  [eV] | **∆*E*_3_**  [eV] | **EQE_EL_**  [×10^-4^] |
| --- | --- | --- | --- | --- | --- | --- | --- | --- |
| D18:BTP-γ-Br | 1.430 | 0.931 | 0.499 | 1.162 | 0.268 | 0.051 | 0.180 | 8.20 |
| D18:BTP-δ-Br | 1.406 | 0.892 | 0.514 | 1.140 | 0.266 | 0.054 | 0.194 | 4.59 |
| D18:N3 | 1.381 | 0.848 | 0.533 | 1.117 | 0.264 | 0.034 | 0.235 | 0.91 |
| D18:N3:BTP-γ-Br | 1.396 | 0.869 | 0.527 | 1.131 | 0.265 | 0.047 | 0.215 | 2.04 |
| D18:N3:BTP- δ -Br | 1.387 | 0.856 | 0.531 | 1.122 | 0.265 | 0.034 | 0.232 | 1.03 |

^a^The optical bandgap (*E*_g_) was determined from the derivatives of the EQE curve.

**Table S8.** The hole and electron mobility of binary and ternary devices.

| **Devices** | *μ*_h_^a^  [×10^-4^ cm^2^ V^-1^ s^-1^] | *μ*_e_^a^  [×10^-4^ cm^2^ V^-1^ s^-1^] | *μ*_h_/ *μ*_e_ |
| --- | --- | --- | --- |
| D18:BTP-γ-Br | 1.78 ± 0.13 | 6.53 ± 0.14 | 0.27 |
| D18:BTP-δ-Br | 0.83 ± 0.02 | 6.25 ± 0.34 | 0.13 |
| D18:N3 | 4.29 ± 0.45 | 9.37 ± 0.18 | 0.46 |
| D18:N3:BTP-γ-Br | 7.33 ± 0.32 | 8.43 ± 0.24 | 0.87 |
| D18:N3:BTP-δ-Br | 6.34 ± 0.54 | 8.07 ± 0.08 | 0.79 |

^a^The average *μ*_h_ and *μ*_e_ in parentheses for 4 devices.

**Table S9.** The detailed GIWAXS data in the out-of-plane direction of blend films.

| **Materials** | **(010) peaks** | | | |
| --- | --- | --- | --- | --- |
|  | ***q***  (Å^-1^) | **d-spacing**  (Å) | **FWHM**  (Å^-1^) | **CCL**  (Å) |
| D18:BTP-γ-Br | 1.715 | 2.66 | 0.278 | 20.3 |
| D18:BTP-δ-Br | 1.708 | 3.68 | 0.306 | 18.5 |
| D18:N3 | 1.738 | 3.61 | 0.241 | 23.5 |
| D18:N3:BTP-γ-Br | 1.734 | 3.62 | 0.250 | 22.6 |
| D18:N3:BTP-δ-Br | 1.730 | 3.63 | 0.255 | 22.2 |

**Table S10.** The detailed GIWAXS data in the in-plane direction of neat films.

| **Materials** | **(100) peaks** | | | |
| --- | --- | --- | --- | --- |
|  | ***q***  (Å^-1^) | **d-spacing**  (Å) | **FWHM**  (Å^-1^) | **CCL**  (Å) |
| D18:BTP-γ-Br | 0.316 | 19.9 | 0.0728 | 77.6 |
| D18:BTP-δ-Br | 0.311 | 20.2 | 0.0745 | 75.9 |
| D18:N3 | 0.299 | 21.0 | 0.0777 | 72.8 |
| D18:N3:BTP-γ-Br | 0.304 | 20.6 | 0.0784 | 72.1 |
| D18:N3:BTP-δ-Br | 0.304 | 20.6 | 0.0841 | 67.2 |
